# Matrix mechanical remodeled carrier-free nanosystem for programmable closed-loop reversal of liver fibrosis via STING alkylation

**DOI:** 10.1126/sciadv.adz4126

**Published:** 2025-11-19

**Authors:** Hongyun Han, Dongrun Yu, Yuxiang Liu, Huizhen Jia, Wenguang Liu

**Affiliations:** ^1^ School of Materials Science and Engineering, Tianjin Key Laboratory of Composite and Functional Materials, Tianjin University, Tianjin 300350, China.; ^2^ State Key Laboratory of Precious Metal Functional Materials, Tianjin University, Tianjin 300350, China.

## Abstract

Extracellular matrix (ECM) sclerosis represents a prominent feature of fibrotic disorders; however, the macrophage response to changes in matrix stiffness and its impact on fibrotic diseases remain unclear. This study reveals a vicious circle of ECM-cell-ECM, where increased ECM hardness activates the STING pathway in macrophages, in turn activates hepatic stellate cells (HSCs), thus enhancing ECM stiffness again and exacerbating liver fibrosis. To reverse liver fibrosis, an innovative carrier-free nanosystem capable of degrading ECM, specifically blocking the STING pathway in macrophages as well as remodeling matrix mechanical, is created. In mouse models, pharmacological STING inhibition via alkylation in macrophages, combined with ECM degradation via matrix metalloproteinases and metal ion–induced macrophage polarization, reduces stromal stiffness and reverses fibrosis. Our findings underscore the antifibrotic potential of matrix mechanical remodeling, demonstrating that concurrent reduction of matrix stiffness and inhibition of STING pathway in macrophages can synergistically promote fibrosis regression. This research establishes a previously unidentified paradigm for liver fibrosis reversal.

## Teaser

A matrix mechanical remodeled carrier-free nanosystem was designed to reverse liver fibrosis through an ECM-cell-ECM closed-loop.

## INTRODUCTION

Excessive accumulation of extracellular matrix (ECM) is a hallmark of liver fibrosis (*1*). The activation of hepatic stellate cells (HSCs) has long been recognized as a primary source of ECM and a crucial factor in the progression of liver fibrosis (*2*). Now, most research has focused on targeting HSCs to mitigate liver fibrosis by modulating the expression of inflammatory factors and other related biochemical mechanisms (*3*–*5*). Although some advancements have been achieved, the therapeutic effect remains uncertain.

In recent years, the rapid development of biomechanics has drawn researchers’ attention to the relationship between biomechanical mechanisms and the onset and development of liver fibrosis. Du *et al.* demonstrated that during the vascularization of hepatic sinusoidal endothelial cells, collagen fibers can be “pulled” along with the migration of these cells when they are within a specific range of stiffness (*6*). The mechanical forces exerted on collagen fibers activate HSCs, leading to increased collagen secretion and creating a vicious cycle that contributes to liver fibrosis (*4*). This underscores the notable impact of mechanical mechanics on the progression of liver fibrosis.

Macrophages are critical effector cells involved in both the progression and resolution of fibrosis (*7*–*9*). As reported in our previous work, there is cross-talk between HSCs and macrophages, and their bidirectional regulation is conducive to the efficient reversal of liver fibrosis (*10*). Many studies have also shown that macrophages drive ECM deposition by fibroblasts during the repair process and can directly degrade and remodel the ECM during resolution (*11*, *12*). It also revealed that the macrophages may have evolved to sense ECM mechanics, allowing them to detect substantial changes in ECM stiffness during tissue repair, thereby serving as proxies to monitor the progression of repair and prevent fibrosis (*13*). However, the response of macrophages to changes in matrix stiffness and its effects on fibrotic diseases remains unclear.

The mechanosensitive channel Piezo1 has been demonstrated to directly sense matrix stiffness (*14*). The Piezo1 pathway in macrophages was shown to be activated by mechanical force, leading to a considerable influx of calcium ions (Ca^2+^), which in turn induces inflammation, activates HSCs, and promotes the development of liver fibrosis (*15*). Mitochondria, as critical regulators of intracellular Ca^2+^ concentration, maintain Ca^2+^ homeostasis through the absorption and release of Ca^2+^. When mitochondrial function is compromised, Ca^2+^ homeostasis is disrupted, thereby affecting the transmission of inflammation-related signals and the expression of cellular immune function (*16*–*18*). Karin *et al.* have shown that Ca^2+^ overload can induce mitochondrial activation and the release of mitochondrial DNA (mtDNA) (*19*). The cyclic GMP-AMP synthase (cGAS)-stimulator of interferon genes (STING) pathway is a crucial component of innate immunity, functioning as a defense mechanism. It can recognize mtDNA released during liver injury, leading to its activation and the triggering of inflammation with the production of type I interferons (IFNs) and proinflammatory cytokines, which exacerbates liver inflammation and hepatocyte damage (*20*–*22*). Several reports have indicated that the STING is highly expressed on macrophages and is involved in the pathogenesis of various fibrotic diseases, including pulmonary fibrosis, liver fibrosis, and so on (*23*). However, the relationship between macrophage STING signaling and ECM stiffness remains elusive.

**Fig. 1.**
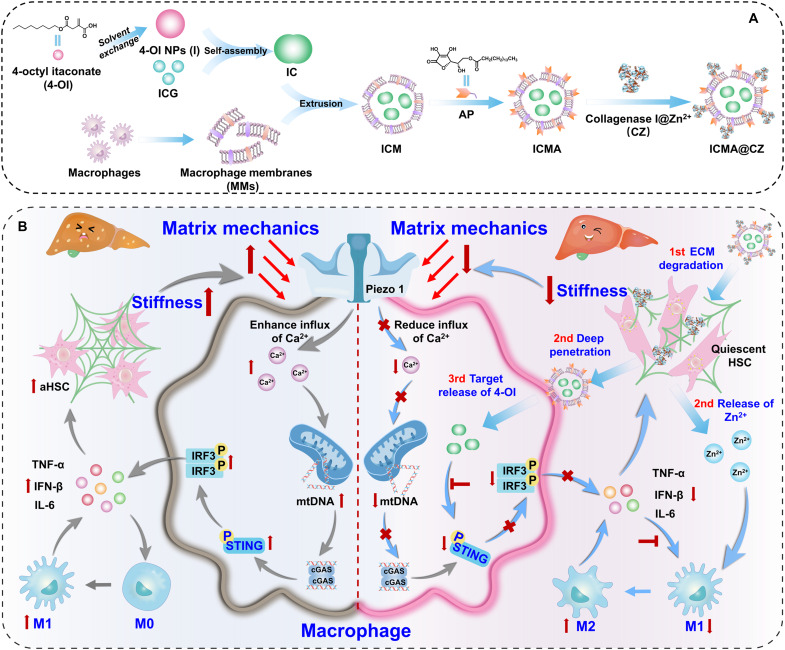
Nanosystem design and programmed closed-loop reversal of liver fibrosis. (**A**) Preparation of MMs and matrix mechanics remodeled carrier-free nanosystem (ICMA@CZ) by packing the 4-OI and ICG coassembly nanoparticles with MMs modified with matrix metalloproteinase (CZ). (**B**) Schematic illustration of macrophage homologous targeting the carrier-free nanosystem loaded with CZ to degrade ECM efficiently while in parallel released 4-OI to block the STING pathway in macrophage, combining Zn^2+^ to regulate the polarization of macrophages and remodel the matrix mechanics to achieve a programmed closed-loop reversal of liver fibrosis through modulating the ECM-cell-ECM circuit. aHSC, activated hepatic stellate cells. NPs, nanoparticles.

Here, we hypothesized that there is an ECM-cell-ECM circuit, in which macrophages sense changes in ECM stiffness, leading to the influx of Ca^2+^ and mitochondria damage, which in turn activates the STING pathway and subsequently stimulates HSCs through the release of proinflammatory cytokines. This process increases the mechanical strength of the ECM and creates a deteriorated circulation of ECM-cell-ECM, ultimately resulting in the deterioration of liver fibrosis. We envision that a carrier-free nanosystem containing matrix metalloproteinase (CZ) and STING inhibitor [4-octyl itaconate (4-OI)] is a promising resolution to reverse the fibrosis. Simultaneously, the CZ was loaded through the coordination interaction between zinc ions (Zn^2+^) and hydroxyl groups. We propose that matrix hardness can be remodeled through ECM degradation mediated by CZ, which enhances the deep penetration of nanoparticles into liver tissue. Further, the macrophage-derived membrane-coated carrier-free nanosystem can achieve highly specific self-recognition to resident macrophages in livers. The release of 4-OI can block the activation of STING pathway in homologous targeted macrophages, while the polarization of macrophages can be regulated by Zn^2+^. This results in the down-regulation of the inflammatory factors expression, thereby blocking the activation of HSCs, reducing the production of ECM and further remolding the mechanical properties of the matrix. Ultimately, a programmed closed-loop reversal of liver fibrosis can be achieved (Fig. 1). Together, this study suggests that remodeling the mechanical microenvironment and regulating the ECM-cell-ECM circuit represent an innovative approach for the treatment of liver fibrosis.

## RESULTS

### Macrophages sense matrix mechanics to control liver fibrosis associated pathways

**Fig. 2.**
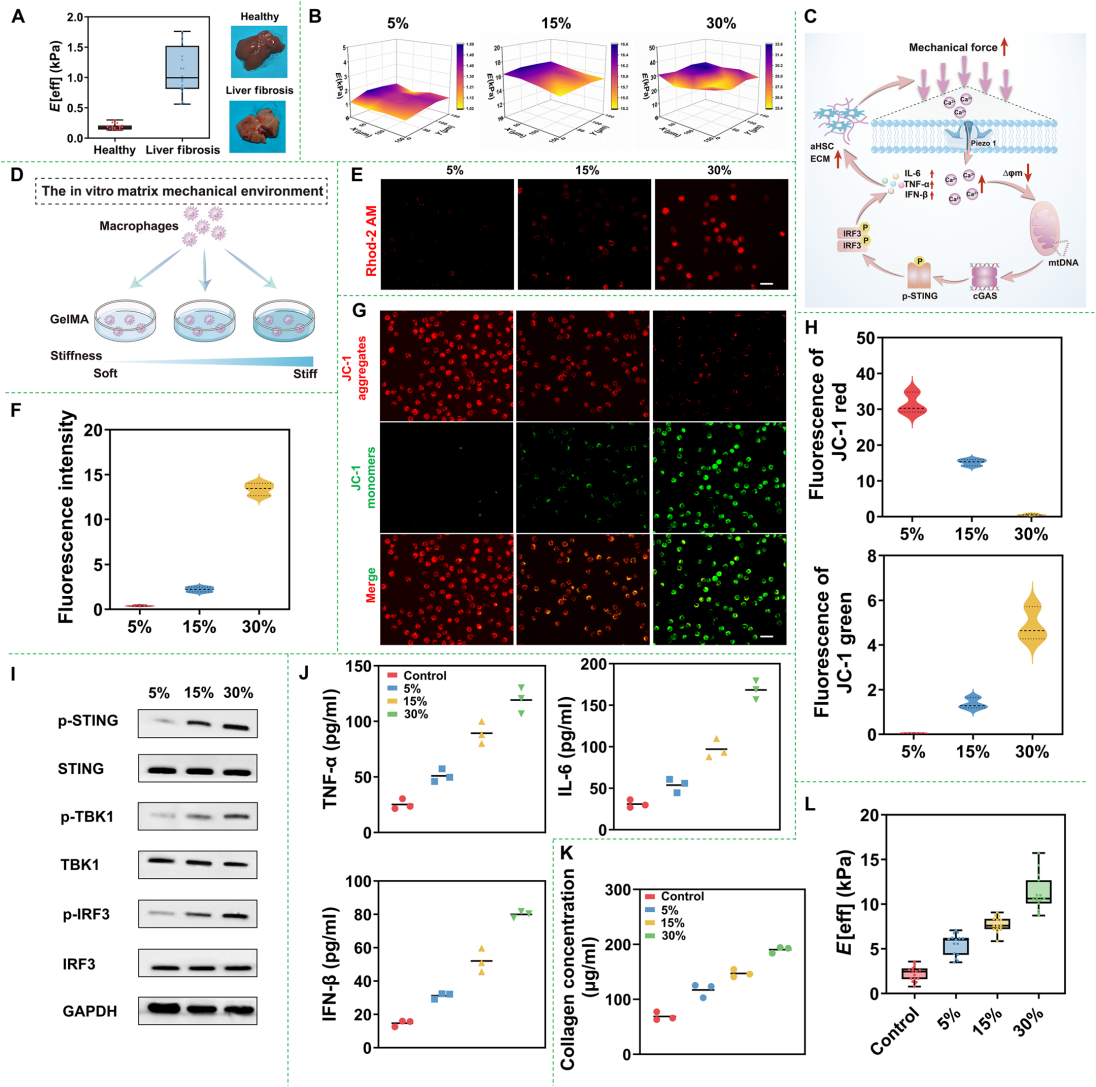
Reveal of the ECM-cell-ECM vicious circle. (**A**) Mechanical strength and photograph of liver tissues with/without liver fibrosis. (**B**) Three-dimensional surface plots display surface hardness uniformity of hydrogels. (**C**) The response of macrophages to changes in matrix stiffness through a cycle of ECM-cell-ECM. Mechanism mechanics induce influx of Ca^2+^, mitochondrial damage, STING pathway activation, secretion of proinflammatory factors, HSC activation, and further resulting enhancement of matrix stiffness. (**D**) Approach to model the in vitro matrix mechanical environment. (**E**) Microphotographs of intracellular Ca^2+^ production of RAW 264.7 cells after treatments with various concentrations of GelMA hydrogel for 24 hours and (**F**) mean fluorescence intensity (MFI) of intracellular Ca^2+^ were analyzed using ImageJ software. (**G**) Mitochondrial membrane potentials of RAW 264.7 cells treated with various concentrations of GelMA hydrogel for 24 hours and (**H**) MFI of mitochondrial membrane potentials in RAW 264.7 were analyzed using ImageJ software. (**I**) Western blot detection of STING, TBK1, IRF3, p-STING, p-TBK1, and p-IRF3 in RAW 264.7 cells subjected to various concentrations of GelMA hydrogel treatments. (**J**) ELISA assay for extracellular secretion of TNF-α, IL-6, and IFN-β by RAW 264.7 cells at 24 hours after different treatments (*n* = 3). (**K**) HSCs were treated with CM from RAW 264.7 cells stimulated by various concentrations of GelMA hydrogel for 24 hours. Subsequently, total collagen was quantified using Sirius Red assay (*n* = 3). (**L**) Mechanical strength of HSC-T6 cells treated with CM from RAW 264.7 cells stimulated by various concentrations of GelMA hydrogel for 24 hours. GAPDH, glyceraldehyde phosphate dehydrogenase.

To model a simple, well-defined matrix mechanical environment, gelatin methacryloyl (GelMA) hydrogels with a weight ratio from 5% (soft) to 30% (stiff) were synthesized (figs. S1 and S2). The change in concentration could alter the stiffness of the GelMA hydrogel, which varied from an elastic modulus of 0.1 to 5 kPa (fig. S3), a range consistent with healthy liver tissue to fibrotic liver tissue (0.1 to 1.8 kPa) (Fig. 2A) (*24*). In addition, the hydrogel exhibited relatively homogeneous surface hardness (Fig. 2B), making it suitable for use as a simulated matrix microenvironment for cell culture. To determine the response of macrophages to changes in matrix stiffness (Fig. 2C), a murine macrophage cell line (RAW 264.7) was selected and cultured within hydrogels of varying stiffness (Fig. 2D). Subsequently, the cells were harvested and stained with a mitochondrial Ca^2+^ indicator (Rhod-2 AM) at room temperature for 30 min. As shown in Fig. 2E, the red fluorescence in macrophages, which represents the concentration of Ca^2+^, was markedly increased after culturing the cells in the 30% GelMA hydrogel (5 kPa). Quantitative data further demonstrated that the concentration of Ca^2+^ in macrophages treated with 30% GelMA hydrogel increased by approximately 15-fold compared to that in the initial cells (Fig. 2F).

To assess mitochondrial Ca^2+^ overload, mitochondrial membrane potentials of RAW 264.7 cells treated with different concentrations of GelMA hydrogel were measured using a JC-1 probe. Normally, the mitochondria in RAW 264.7 cells maintained a high mitochondrial membrane potential, causing JC-1 to exhibit red fluorescence due to the formation of J-aggregates. Conversely, at a low mitochondrial membrane potential, JC-1 showed green fluorescence due to the presence of JC-1 monomers (*25*). As illustrated in Fig. 2G, all cells treated with varying concentrations of GelMA hydrogel displayed lower mitochondrial membrane potentials compared to the control group. Notably, RAW 264.7 cells in the group treated with 30% GelMA hydrogel exhibited the highest intensity of green fluorescence, which was five to seven folds higher than that in the cells cultured with 5 and 15% GelMA hydrogel, respectively (Fig. 2H), indicating the lowest mitochondrial membrane potential and the most severe mitochondrial damage. Furthermore, the cGAS-STING pathway in macrophages was activated in conjunction with the increasing concentration of GelMA hydrogel, which primarily manifested as elevated levels of corresponding protein phosphorylation including phospho-stimulator of interferon genes (p-STING), phospho-TANK-binding kinase 1 (p-TBK1), and phospho-interferon regulatory factor 3 (p-IRF3) (Fig. 2I). Concurrently, the production of proinflammatory cytokines, including tumor necrosis factor–α (TNF-α), IFN-β, and interleukin-6 (IL-6), was also enhanced with increasing GelMA hydrogel concentration (Fig. 2J). After immortalized rat liver stellate cells (HSC-T6) were treated with the conditioned medium (CM) from RAW 264.7 stimulated with varying concentrations of GelMA hydrogel for 24 hours, a substantial increase in collagen content was observed in the HSC-T6 cells. Notably, greater hydrogel stiffness correlated with higher collagen levels (Fig. 2K). Meanwhile, the matrix mechanics were assessed using a Chiaro Nanoindenter. As shown in Fig. 2L, with increasing hydrogel concentration, matrix mechanics also increased, aligning with the changes in collagen content. This supported our hypothesis that the vicious cycle of ECM-cell-ECM ultimately led to the progression of liver fibrosis. Therefore, remodeling the ECM and reducing the secretion of proinflammatory factors could be crucial steps in reversing the process of liver fibrosis.

### Preparation and characterization of carrier-free nanosystem

To verify that the inhibition of the ECM-cell-ECM vicious cycle can induce the reversal of liver fibrosis, a matrix mechanical remodeled carrier-free nanosystem was synthesized. The 4-OI carrier-free nanoparticles were first synthesized via a reprecipitation method (*26*). To impart fluorescence indication functionality to the carrier system, amphipathic indocyanine green (ICG), which has the potential to detect liver fibrosis (*27*), was coassembled into the 4-OI nanoparticles, referred to as IC. Considering the homologous targeting characteristics of cell membranes, the mouse macrophage cell line RAW 264.7 was used to generate the cracked macrophage membranes (MMs) using a membrane protein extraction kit and differential centrifugation. The MM-coated nanosystem was prepared by mixing the MMs with IC nanoparticles. To streamline the research, the mass ratio of cell membrane versus IC nanoparticles was first optimized on the basis of dynamic light scattering (DLS) assay. As shown in fig. S4, the hydrodynamic sizes of the IC and MMs were 110 and 200 nm, respectively. When the mass ratios of MMs versus IC were fixed at 3:1 and 4:1, the mean hydrodynamic diameter (*D*_h_) of nanoparticles was around 120 nm, and the zeta potential of the nanoparticles at 3:1 was nearly identical to that of MMs. To avoid the excessive MM presence, the mass ratio of MMs versus IC was fixed at 3:1. In addition, there was minimal change in the *D*_h_ after standing at room temperature for 24 hours (fig. S5), suggesting that the MMs were almost completely coated on the surface of IC nanoparticles. Thus, the nanosystem was designated as ICM for further study. To endow the nanosystem with matrix mechanical remodeling properties, the matrix metalloproteinases (CZ) were introduced onto the nanosystem surface through the complexation of ascorbyl palmitate (AP) and metal ions. To optimize the mass of AP and CZ, DLS assay was used. As illustrated in fig. S6, the size of AP-modified nanoparticles (ICMA) initially decreased, then increased, and subsequently experienced a slight decrease with increasing AP dosage. The nonlinear size variation of ICMA may be attributed to the dynamic assembly between the AP and the nanoparticles. Initially, the carbon chains of AP inserted into the cell membrane and assembled with the IC through hydrophobic interactions, which compressed the particle size. As the concentration of AP increased, the AP self-assembled on the surface of cell membrane, resulting in an increase in particle size. With further addition of AP, the insertion of AP on the cell membrane surface reached saturation, leading to the formation of small-sized AP self-assemblies in the solution, which caused a slight decrease in the average particle size of the system (*28*). At an AP concentration of 8 μg, the diameter of ICMA was nearly the same as that of ICM, and the zeta potential was comparable to that of AP. To avoid excessive AP existence, 8 μg of AP was used to perform the further study. Similarly, when the mass of CZ was 250 μg, the diameter of CZ-modified nanoparticles (ICMA@CZ) was less than 200 nm, with a zeta potential of −20 mV. An increase in CZ concentration resulted in an increase in the diameter of ICMA@CZ. In addition, the data indicated that when the concentration of CZ exceeded 350 μg, the zeta potential reached a plateau. However, larger particles (>200 nm) were clearly observed (fig. S7), with the polydispersity index of the nanoparticles being approximately 0.5. The literature has shown that nanoparticles with a diameter greater than 200 nm pose a risk of activating the complement system. Particles with diameters of 50 and 200 nm can escape from the splenic space, enter the sinusoidal endothelial pores, and accumulate in large quantities at sites of liver fibrosis (*29*, *30*). To ensure the reliability of the nanoparticles and to prevent the presence of excessive CZ, the mass of CZ was fixed at 250 μg and used for subsequent studies.

**Fig. 3.**
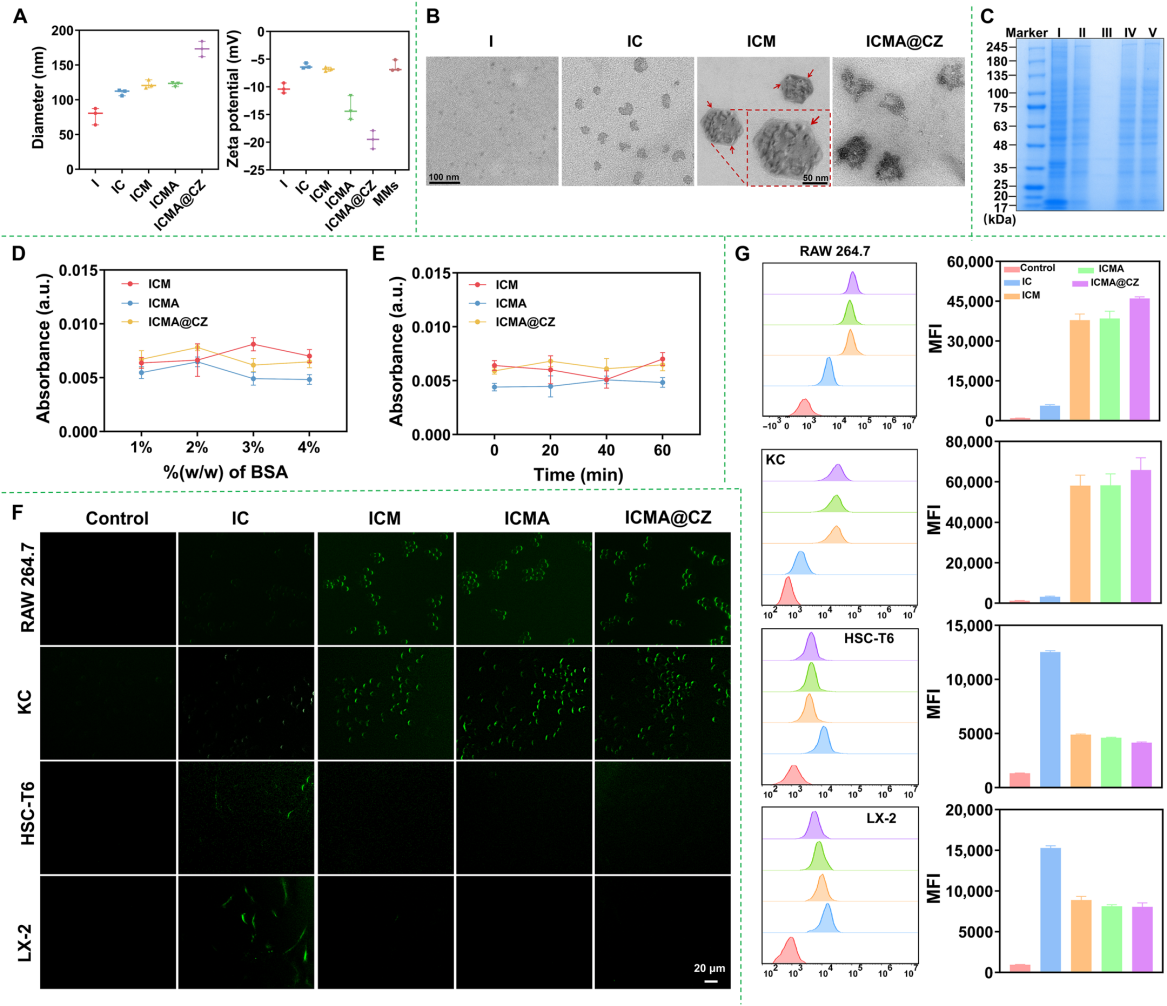
Synthesis and characterization of carrier-free nanosystems. (**A**) Particle sizes and zeta potentials of I, IC, ICM, ICMA, and ICMA@CZ; the zeta potential of MMs used as a control. (**B**) TEM images of I, IC, ICM, and ICMA@CZ. (**C**) SDS–polyacrylamide gel electrophoresis protein analysis of macrophage cell membrane. Samples were stained with Coomassie Blue. I) source macrophage cell membrane, II) MMs, III) IC, IV) ICM, and V) ICMA. (**D**) Absorbance of ICM, ICMA, and ICMA@CZ after incubation with varying ratios of BSA for 1 hour. (**E**) ICM, ICMA, and ICMA@CZ absorbance at 350 nm after different incubation times with 4% BSA. (**F**) Microphotographs of four cell lines including RAW 264.7, KC, HSC-T6, and LX-2 after incubation for 1 hour with IC, ICM, ICMA, and ICMA@CZ. Scale bar, 20 μm. (**G**) Flow cytometry analysis of fluorescence intensities inside four cells after treated by different samples for 1 hour and MFI were analyzed using FlowJo software. The concentration of ICG was fixed at 2 μg ml^−1^. a.u., arbitrary units.

The size and zeta potential of I, IC, ICM, ICMA, and ICMA@CZ were presented in Fig. 3A. The hydrodynamic sizes of the I and IC were around 80 and 110 nm, respectively. The IC nanoparticles were formed through strong hydrophobic interactions and π-π stacking between ICG and carrier-free 4-OI nanoparticles, which were fabricated from the aggregation of hydrophobic 4-OI molecules. After the addition of the MMs, the hydrodynamic size of ICM increased to 125 nm with a polydispersity of 0.100 to 0.180, since one or more IC nanoparticles can be encapsulated by MMs (*26*, *31*). The zeta potential of ICM was measured at −7 mV, which was comparable to that of MMs (−7 mV) but lower than that of IC (−6 mV). With the introduction of AP, the zeta potential decreased slightly by −14 mV for the assembly of AP on the surface of MMs (*28*). Following the modification with CZ, the zeta potential of ICMA markedly dropped to −20 mV (Fig. 3A). Transmission electron microscopy (TEM) revealed a superficial coverage of the MM layer. The *D*_h_ of ICM was closely aligned with the mean particle size measured by TEM in a dry state (Fig. 3B), indicating the compact coverage of MMs in an aqueous medium. Furthermore, the TEM image of ICMA@CZ demonstrated the successful loading of CZ onto the surface of ICMA (Fig. 3B and fig. S8). The mean particle size measured by TEM in a dry state was also consistent with the *D*_h_ of ICMA@CZ. In addition, the loading efficiency of 4-OI in the nanosystems was estimated to be ~96%, which was quantified using high-performance liquid chromatography (HPLC) at a wavelength of 210 nm.

Subsequently, gel electrophoresis was performed to investigate the transfer of the membrane protein from the macrophage cell membrane to the shells of ICM. The results indicated that the ICM, ICMA, and MMs exhibited nearly identical protein profiles when compared with RAW 264.7 cell lysate. Obviously, proteins from the cell membrane were effectively retained within the ICM after a series of treatments (Fig. 3C). Ultraviolet-visible spectrophotometry was used to assess the in vitro stability of ICM, ICMA, and ICMA@CZ at 350 nm. As illustrated in Fig. 3 (D and E), both MM-coated nanoparticles displayed minimal serum adsorption behaviors dependent on the bovine serum albumin (BSA) concentration and incubation time. Phosphate-buffered saline (PBS) with 10% fetal bovine serum (FBS) as the physiologically relevant medium was further used to demonstrate the stability of nanoparticles. As shown in fig. S9, the encapsulation of MMs resulted in a highly stable dispersion of nanoparticles, with minimal size changes even after standing at room temperature for 24 hours. In contrast, the size of IC increased to more than twice within 12 hours. The results were consistent with that observed in BSA solution. This emphasizes the ability of negatively charged ICM, ICMA, and ICMA@CZ to maintain stability and preserve their initial characteristics.

To validate our assumption that MM coating would be self-recognized by homotypic macrophage cells, the internalization of MM-coated nanoparticles was assessed in four different cell lines: RAW 264.7 cells, Kupffer cells (KCs), HSC-T6 cells, and human HSCs (LX-2) upon 4-hour coincubation. An amazing outcome was found that the fluorescence intensity of ICG from the MM-coated nanoparticles was markedly higher in macrophage cells (RAW 264.7 cells and KCs) compared to HSCs (HSC-T6 and LX-2 cells) (Fig. 3F). With MM coating, the fluorescence intensity of ICG in RAW 264.7 cells and KCs was approximately 8 to 9 folds and 10 to 12 folds stronger than that in the other cell lines, as determined by quantitative flow cytometry analysis (Fig. 3G). Meanwhile, the IC group displayed weaker green fluorescence intensity in RAW 264.7 cells and KCs, suggesting a highly specific self-recognition affinity of MM-coated nanoparticles for homologous cells, which mitigated the repulsive effects of the negatively charged surface due to homotypic binding properties. These results indicated that the cell membrane coating played a critical role in cellular uptake of the IC nanoparticles. Notably, in the IC group, the fluorescence intensity in HSCs was relative stronger than that in macrophages. This discrepancy may be attributed to the distinct endocytic mechanisms of the two cell types. The literature indicates that the mechanism by which macrophages capture nanoparticles primarily involves receptor-ligand binding rather than nonspecific pathways. Furthermore, macrophages exhibit a considerable capacity to recognize and eliminate larger nanoparticles, whereas their endocytic ability for smaller particles is comparatively weaker (*32*–*34*). In contrast, IC nanoparticles lacked specific targets for macrophages to recognize and were ~100 nm in size, which likely contributed to the unexpected trend observed. In addition, 3-(4,5-dimethyl-2-thiazolyl)-2,5-diphenyl-2-H-tetrazolium bromide (MTT) assay showed that the ICMA@CZ exhibited substantially enhanced biocompatibility in all cell lines including RAW 264.7, HSC-T6, and LX-2 cells, even at the concentration of 1 mg/ml (fig. S10), demonstrating that ICMA@CZ had good biocompatibility and potential for clinical application.

### Matrix mechanical remodeling through ECM degradation

**Fig. 4.**
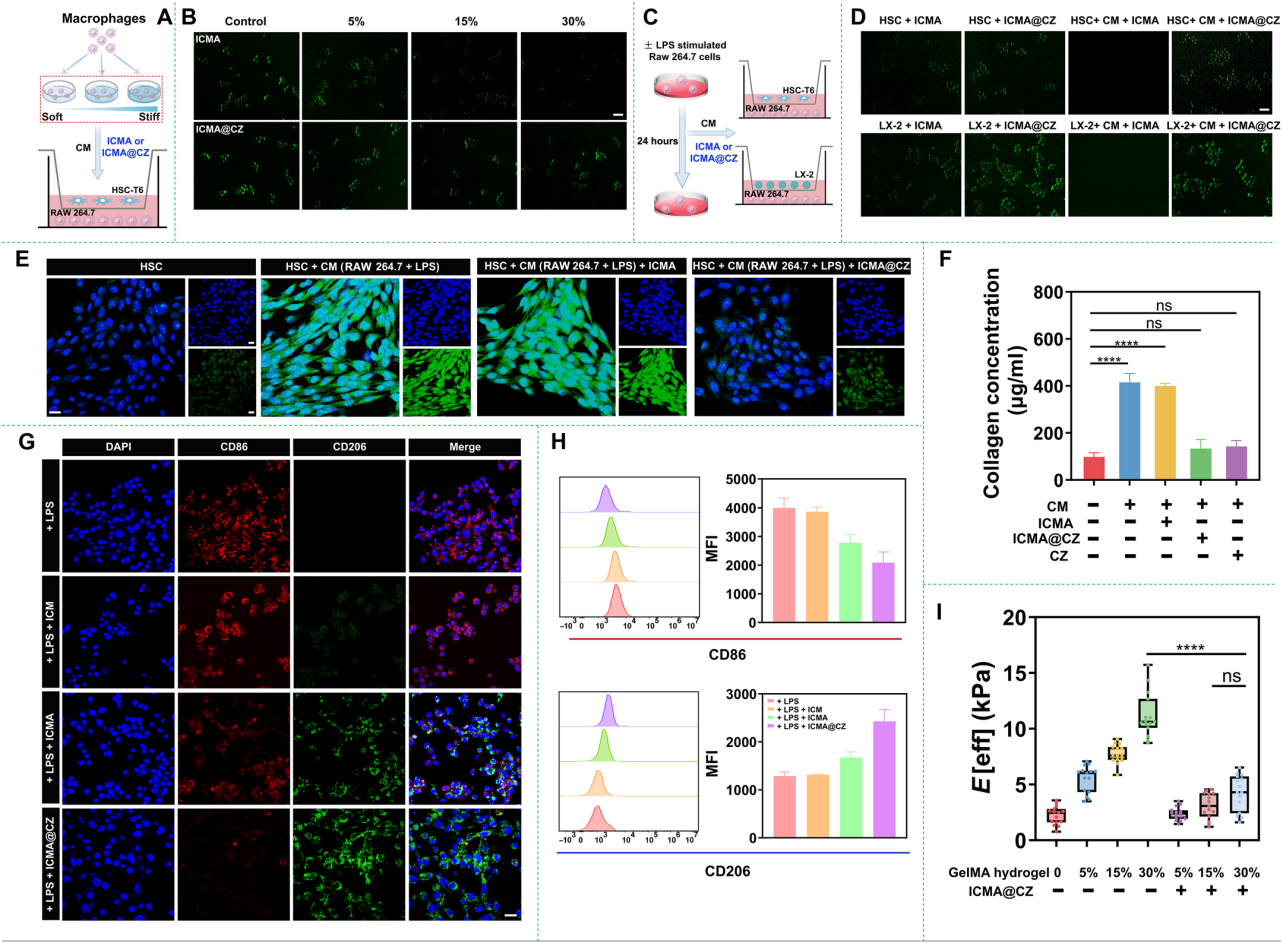
Matrix mechanical remodeling through ECM degradation mediated by carrier-free nanosystems. (**A**) Approach to in vitro analysis of the interactions between matrix mechanics mediated ECM and endocytosis of nanoparticles. (**B**) Microphotographs of RAW 264.7 with adding of ICMA or ICMA@CZ for 2 hours after cocultured with HSC-T6 cells using a transwell plate. The HSC-T6 cells were pretreated with/without the CM from RAW 264.7 cells with/without stimulation of hydrogels (0.1 to 5 kPa) for 24 hours. Scale bar, 20 μm. (**C**) Approach to in vitro analysis of the ability of ICMA@CZ to degrade ECM. (**D**) Microphotographs of RAW 264.7 with adding of ICMA or ICMA@CZ for 2 hours after cocultured with HSC-T6 and LX-2 cells. The HSC-T6 and LX-2 cells were treated with/without the CM from RAW 264.7 cells with or without stimulation of LPS for 24 hours. Scale bar, 20 μm. (**E**) Confocal laser scanning microscopy (CLSM) images of α-SMA in HSC-T6 cells with/without adding of ICMA or ICMA@CZ for 2 hours. HSC-T6 cells were pretreated with/without the CM of RAW 264.7 cells stimulated with/without LPS for 24 hours. (**F**) Total collagen of HSC-T6 cells treated with different formulations was quantified using Sirius Red assay (*n* = 3). (**G**) CLSM images and (**H**) flow cytometry analysis of markers for M1 and M2 phenotypes in the polarization of RAW 264.7 macrophages before and after treatment with different samples for 2 hours. Green (CD86) and red (CD206). Scale bar, 10 μm. The concentration of nanoparticles was fixed at 2 μg/ml. (**I**) Mechanical strength of HSC-T6 cells treated with CM from RAW 264.7 cells stimulated by various concentrations of GelMA hydrogel for 24 hours before and after addition of ICMA@CZ. ns, not significant; DAPI, 4′,6-diamidino-2-phenylindole.

Excessive deposition of ECM is a key feature of liver fibrosis. The activated HSCs have long been recognized as the primary source of ECM and a central factor in the development of liver fibrosis (*35*). Studies have demonstrated that the hardened ECM network considerably restricts the penetration of therapeutic drugs, ultimately affecting their efficacy (*36*). Thus, the activation of HSCs and the mechanical strength of the matrix were evaluated. As shown in Fig. 4 (A and B), when HSC-T6 cells were cocultured with the CM from RAW 264.7 stimulated by GelMA hydrogel with varying stiffness (0.1 to 5 kPa) for 24 hours, the green fluorescence of ICMA in the RAW 264.7 cells cultured in the lower compartment of the transwell decreased as the stiffness of the GelMA hydrogel increased. In particular, after HSC-T6 cells were cultured with CM from RAW 264.7 stimulated by 30% of GelMA hydrogel, almost no green fluorescence was observed in the macrophages in the lower compartment. However, for the ICMA@CZ, although the fluorescence change trend in macrophages was consistent with that in the group of ICMA, the overall fluorescence intensity was higher than that of ICMA. Even when the hydrogel stiffness was 5 kPa, obvious green fluorescence could still be observed in the macrophages, which was nearly identical to that in the free CZ group (fig. S11A). Quantitative data indicated that the presence of CZ led to the green fluorescence intensity in macrophages being 5 to 10 times higher than that of the ICMA group in the condition of 15 and 30% hydrogel, respectively (fig. S12A). Combining the results in Fig. 2K that the activation of HSC-T6 was positively correlated with the increase in hydrogel concentration, it could be initially demonstrated that ICMA@CZ had an ability to degrade collagen caused by the mechanics of matrix.

To further directly test the ECM degradation ability of ICMA@CZ, lipopolysaccharide (LPS) was used to model a simple, well-defined matrix mechanical environment. As shown in Fig. 4C, the RAW 264.7 cells were first treated by LPS for 24 hours, and the CM was collected to coculture with HSC-T6 using a transwell plate. It was found that the uptake of ICG-labeled nanoparticles in RAW 264.7 cells mediated by ICMA@CZ was higher than that in cells treated with ICMA. Although the cells were not activated by CM, the uptake of nanoparticles in RAW 264.7 cells mediated by ICMA@CZ was still higher than that in cells treated with ICMA, which was nearly the same with that in the free CZ group (fig. S11B). In addition, to test the potential ability of ICMA@CZ used in the clinical, the LX-2 cells were also treated with the CM, and the uptake of nanoparticles mediated by ICMA@CZ in RAW 264.7 cells was analyzed. The results were similar to those observed in the HSC-T6 cells (Fig. 4D and fig. S11C). These findings suggested that the activity of CZ was not influenced by the presence of the nanosystems. Quantitative data showed that the fluorescence in cells treated with ICMA@CZ was almost 12 to 13 folds higher than that in the cells treated with ICMA (fig. S12B). To elucidate the mechanisms involved in the collagen degradation ability of CZ, CM from RAW 264.7 cells, with or without stimulation with LPS at the concentration of 100 ng/ml for 24 hours, was added into the HSC-T6 cells, and after stimulation for 24 hours, the cells were further cultured in the medium containing of ICMA or ICMA@CZ nanoparticles at the concentration of 2 μg/ml for 2 hours. Subsequently, the expression of α-smooth muscle actin (α-SMA) (a marker of activated HSCs) and the ECM degradation efficiency of ICMA@CZ were assessed using immunofluorescent staining. As expected, the green fluorescence of α-SMA in the HSCs was markedly increased under CM conditions, especially with CM from the RAW 264.7 cells stimulated with LPS, demonstrating that the HSC-T6 cells could be activated by the CM from activated RAW 264.7 cells. With the addition of ICMA@CZ, the green fluorescence was noticeably decreased, and α-SMA expression returned to levels nearly identical to those of quiescent cells. Quantitative data showed that the fluorescence in cells treated with ICMA@CZ was approximately three to four folds lower than that in the activated HSC-T6 cells (Fig. 4E and fig. S13).

Similarly, collagen serves as another important marker of activated HSCs, which was assessed using the Sirius Red Total Collagen Detection Assay Kit. As depicted in Fig. 4F, the addition of ICMA@CZ resulted in a marked reduction of collagen levels, comparable to those observed in the free CZ group and approaching the levels seen in quiescent HSCs. In contrast, there was minimal change observed in the ICMA group. Previous studies have reported that M1 macrophages promote ECM deposition (*37*). In addition, some reports indicated that zinc ions (Zn^2+^) notably influence M2 macrophage polarization, which is associated with anti-inflammatory responses and ECM remodeling (*38*). This prompted us to investigate the ability of ICMA@CZ to regulate macrophage polarization and facilitate the remodeling of the matrix’s mechanical microenvironment, as assessed through immunofluorescent staining for CD86 and CD206. The results presented in Fig. 4G and fig. S14 demonstrated that RAW 264.7 cells exhibited increased expression of CD86 under stimulation with LPS, indicative of M1 macrophage polarization. Cotreatment with ICMA@CZ resulted in a remarkable increase in the immunofluorescence intensity of the M2 macrophage marker CD206. Flow cytometry analysis indicated that the addition of ICMA@CZ resulted in a 2.5-fold increase in the number of CD206-positive cells compared to the initial state, while the number of CD86-positive cells was decreased by more than twice (Fig. 4H). Collectively, these findings suggested that ICMA@CZ promoted the polarization of RAW 264.7 cells toward M2 macrophages, aligning with previous studies, highlighting the role of zinc ions in macrophage polarization. Furthermore, the mechanical properties of the matrix before and after addition of ICMA@CZ were evaluated using a Chiaro Nanoindenter. As shown in Fig. 4I, the mechanical strength of the matrix was significantly improved with increased hydrogel stiffness. Following the addition of ICMA@CZ, the stiffness of ECM markedly decreased, nearly matching that of the original cells. All the results indicated that ICMA@CZ had the capacity to regulate macrophage polarization and assist in remodeling the mechanical microenvironment of the matrix. However, it was noteworthy that Fig. 4G revealed that costimulation with ICM and ICMA led to a partial reversal in CD86 expression although there were no zinc ions, potentially due to the ability of 4-OI to modulate macrophage polarization via alkylating cysteine sites of STING (*39*).

### Matrix mechanical remodeling by inhibition of STING pathway

**Fig. 5.**
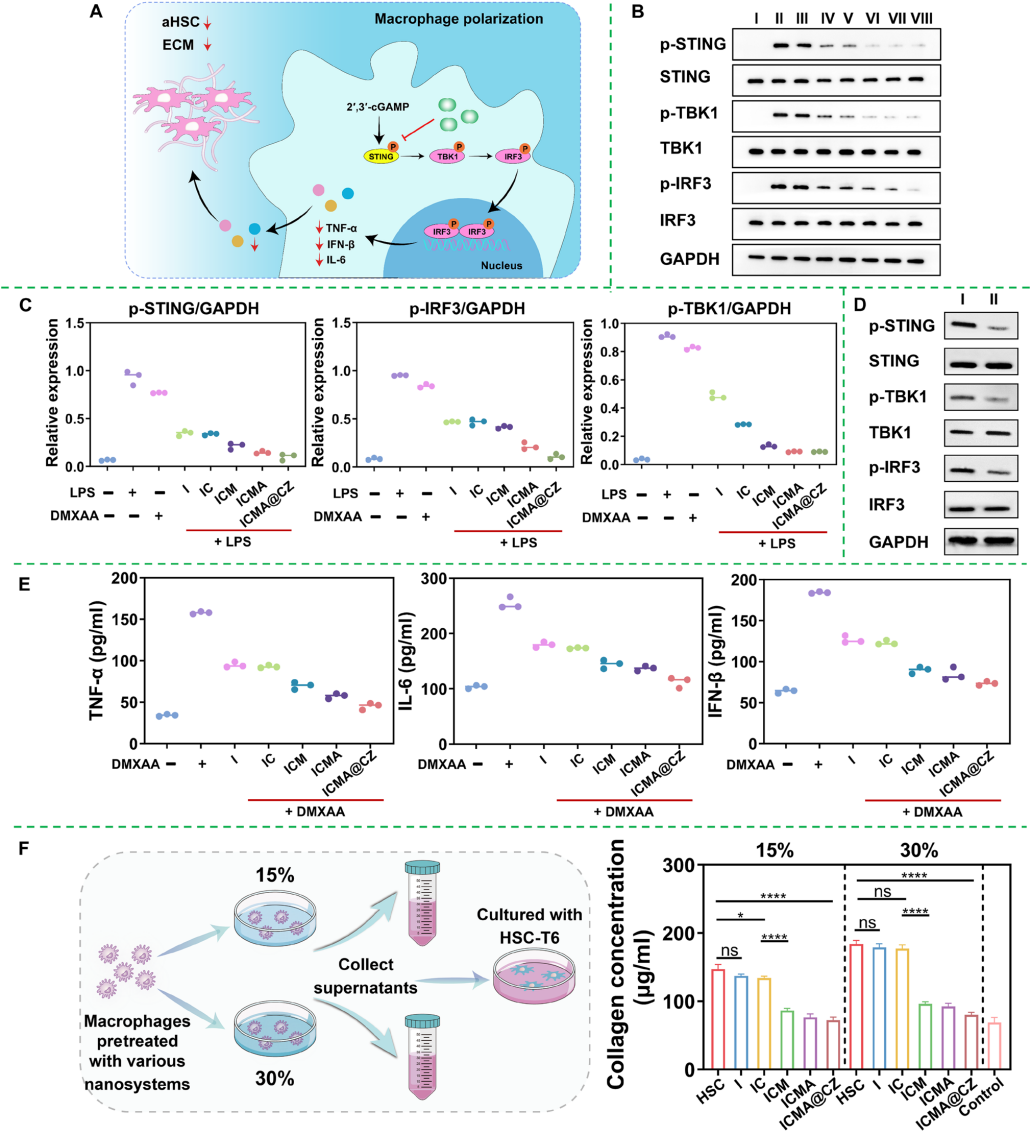
Matrix mechanical remodeling via STING alkylation mediated by carrier-free nanosystem. (**A**) ICMA@CZ remodeled ECM mechanical microenvironment through inhibiting cGAS-STING pathway in vitro. (**B**) Western blot and protein levels of (**C**) p-STING, p-IRF3, and p-TBK1 in RAW 264.7 cells quantified by densitometry and normalized by GAPDH levels (*n* = 3) after different treatments. I: RAW 264.7 cells; II: RAW 264.7 + LPS; III: RAW 264.7 + DMXAA; IV: RAW 264.7 + I + LPS; V: RAW 264.7 + IC + LPS; VI: RAW 264.7 + ICM + LPS; VII: RAW 264.7 + ICMA + LPS; VIII: RAW 264.7 + ICMA@CZ + LPS. (**D**) Representative Western blotting analysis of STING, TBK1, and IRF3 and phosphorylation of related proteins, which were extracted from RAW 264.7 cells after different treatments. I: RAW 264.7 + 30% hydrogel; II: RAW 264.7 + ICMA@CZ + 30% hydrogel. (**E**) Production of TNF-α, IL-6, and IFN-β [detected by enzyme-linked immunosorbent assay (ELISA)] in culture media in response to RAW 264.7 cells stimulated by exposure to the various conditions. Value represents mean and SD (*n* = 3). (**F**) Approach to in vitro analysis of the ability of ICMA@CZ to remodel the ECM mechanical microenvironment through inhibiting the STING pathway, and HSC-T6 cells were treated with/without CM from RAW 264.7 pretreated with/without different nanosystems for 2 hours and then stimulated with hydrogels for 24 hours; after that, total collagen was quantified using Sirius Red assay (*n* = 3), and quiescent HSC-T6 cells were used as a control. Differences between groups were statistically significant. Data were analyzed using the Student’s *t* test (****P < 0.0001 versus HSC-T6 or IC group and *P < 0.05 versus HSC-T6 group).

As demonstrated in Fig. 2, the STING pathway in macrophages was activated alongside an increase in matrix mechanics, thereby promoting cellular polarization. To illustrate that the carrier-free nanosystems can inhibit the phosphorylation of STING and thereby blocking its activation in macrophages and remodel the ECM mechanical microenvironment (Fig. 5A), the RAW 264.7 cells were activated using a stiff hydrogel (5 kPa) or LPS. DMXAA was used as an agonist of the cGAS-STING pathway to stimulate RAW 264.7 cells as a positive control. Western blot analysis of proteins STING, TBK1, IRF3, and their phosphorylated forms extracted from RAW 264.7 cells indicated that the expression of p-STING, p-TBK1, and p-IRF3 was inhibited following treatment with I, IC, ICM, ICMA, and ICMA@CZ, with the expression levels of phosphorylated proteins in the I and IC groups being comparable. This suggested that the ability of I to inhibit the STING pathway was unaffected by the introduction of ICG. Because of the targeting of cell membranes, ICM, ICMA, and ICMA@CZ exhibited stronger inhibitory effects on phosphorylated proteins (Fig. 5, B and C). Similarly, the STING pathway in macrophage can also be activated by the stiff hydrogel, while the expressions of p-STING, p-TBK1, and p-IRF3 proteins were effectively inhibited by ICMA@CZ (Fig. 5D). This indicated that ICMA@CZ had the strongest ability to block the activated STING pathway in macrophages.

Since the activated STING pathway was known to secrete TNF-α, IL-6, and IFN-β, the elaboration of these factors was evaluated in the context of 4-OI. DMXAA was used as an agonist of the cGAS-STING pathway to stimulate RAW 264.7 cells. Figure 5E showed that 4-OI–based nanoparticles induced a considerable down-regulation of TNF-α, IL-6, and IFN-β secretion. Particularly in the context of introducing MMs, the concentrations of TNF-α, IL-6, and IFN-β were around three to four folds lower than that of the cells treated with the cGAS-STING pathway agonist, attributed to the homologous targeting of the macrophage cell membrane and enhanced concentration of 4-OI within the cells, which was consistent with the results shown in Fig. 3F. Furthermore, collagen as another important marker of activated HSCs and ECM mechanical microenvironment demonstrated a significant reduction in HSCs treated with CM from activated RAW 264.7 cells, which had been pretreated with ICMA or ICMA@CZ for 2 hours and stimulated with stiff hydrogel for 24 hours. The amount of collagen was reduced to nearly the normal levels observed in quiescent HSCs (Fig. 5F). This set of data suggested that the 4-OI containing carrier-free nanosystem had the capability to block the activation of STING pathway in macrophage stimulated by increased ECM mechanics, thereby inducing down-regulation of inflammatory factor production, inhibiting HSC activation, and further reducing collagen deposition, thus realizing the ECM-cell-ECM closed-loop virtuous cycle.

### In vivo antifibrosis efficacy of the nanosystem

**Fig. 6.**
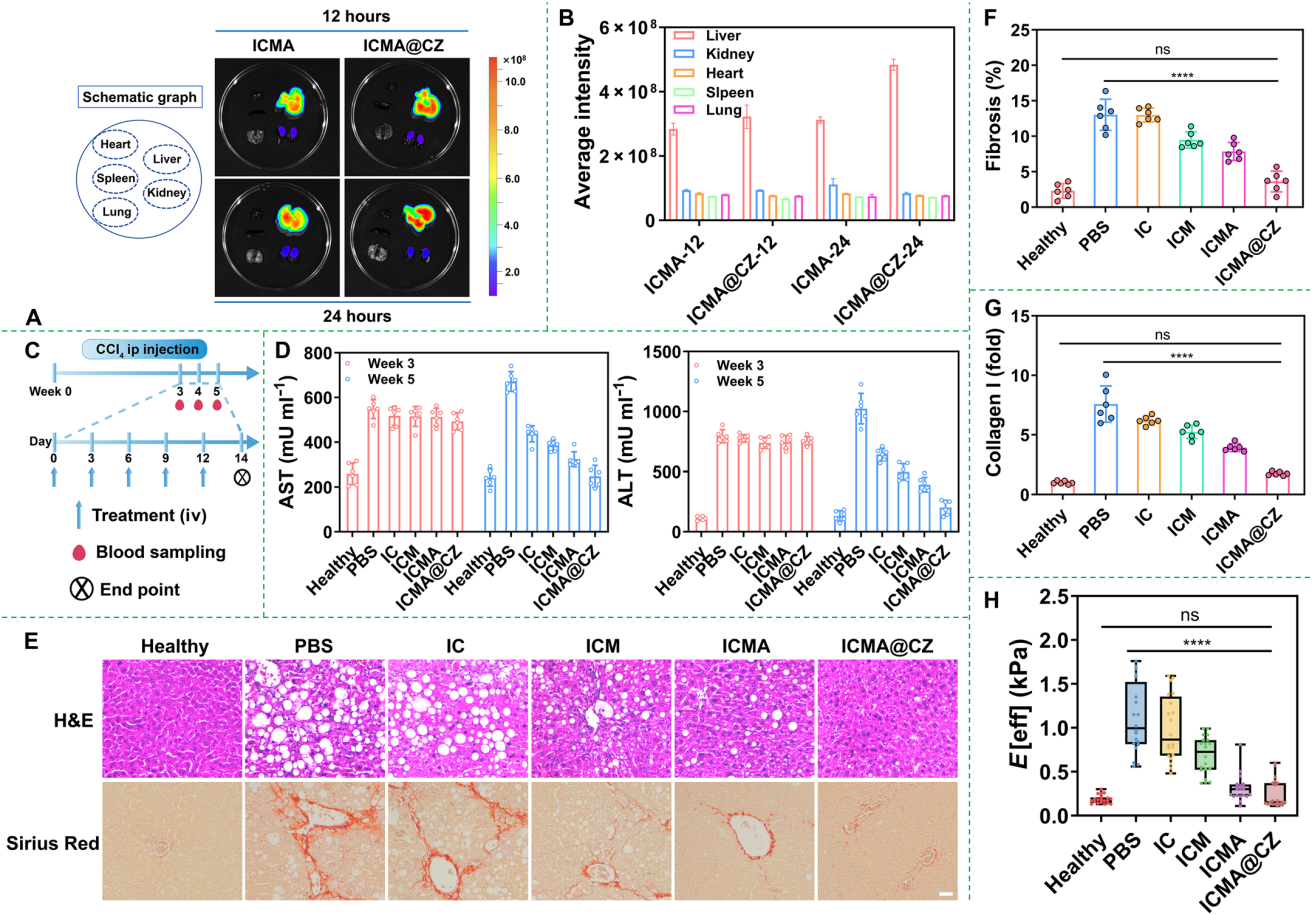
In vivo antifibrosis efficiency and matrix mechanical remodeling ability of carrier-free nanosystems. (**A**) Ex vivo fluorescence imaging of major organs after intravenously injection of ICMA@CZ for 12 and 24 hours. (**B**) Ex vivo quantification of fluorescence distribution in major organs at 12 and 24 hours (*n* = 3, mean ± SD). (**C**) Schematic representation of the CCl_4_-induced liver fibrosis model and treatment timeline for PBS, IC, ICM, ICMA, or ICMA@CZ nanosystem therapy. ip, intraperitoneal; iv, intravenous. (**D**) Liver injury characterization by serum AST and ALT in mice from the PBS, IC, ICM, ICMA, or ICMA@CZ nanosystem treatment groups (*n* = 6). (**E**) Representative hematoxylin and eosin (H&E) staining and immunofluorescence staining of Sirius Red in CCl_4_-induced fibrotic livers from the PBS, IC, ICM, ICMA, or ICMA@CZ nanosystem treatment groups. Scale bar, 100 μm. (**F**) Quantification analysis of fibrosis in CCl_4_-induced fibrotic livers from the PBS, IC, ICM, ICMA or ICMA@CZ nanosystem treatment groups was performed by ImageJ. (**G**) Relative mRNA expression of collagen I in CCl_4_-induced fibrotic livers from the PBS, IC, ICM, ICMA, or ICMA@CZ nanosystem treatment groups (*n* = 6). Healthy mice used as control. (**H**) Mechanical strength of livers after different treatments. Healthy liver used as control. Data were analyzed using Student’s *t* test (****P < 0.0001 versus PBS group).

The encouraging results of in vitro experiments evoked us to evaluate the in vivo biodistribution of nanosystems. Initially, a mouse model with alcohol-associated liver disease (AALD) was intravenously injected with free ICG, IC, ICM, ICMA, or ICMA@CZ, both at an identical ICG dosage of 5 mg kg^−1^. Ex vivo imaging of major organs (heart, liver, spleen, lung, and kidney) was performed at 12 and 24 hours postinjection. As shown in Fig. 6A, the ICMA and ICMA@CZ groups exhibited noticeably stronger fluorescence of ICG in the liver compared to the other organs. In contrast, substantial fluorescence of ICG was observed in the kidneys in the free ICG group. Even in the IC group, the fluorescence in the livers was markedly weaker (fig. S15). This turned out that the ICG diffused out of the IC nanoparticles during blood circulation, resulting in rapid clearance from the body. These findings indicated the efficient liver accumulation of the nanosystems with MM coating. Quantitative analysis of fluorescence intensity in the liver also revealed that the intraliver content of ICG was approximately six folds higher than that in other organs. Particularly for ICMA@CZ at 24 hours, the fluorescence of ICG in the liver was around 1.5 folds greater than that of ICMA (Fig. 6B), demonstrating that the introduction of CZ can overcome the barriers of ECM and achieve deep penetration. Meanwhile, this study also confirmed that the nanosystem can achieve efficient enrichment in the liver due to the homologous targeting of MMs.

To confirm that the enhanced liver accumulation of ICMA@CZ could effectively treat liver fibrosis, the in vivo antifibrosis experiments of ICMA with/without CZ were carried out. An AALD mouse model with moderate liver fibrosis was established by a 5-week adminstration of CCl_4_. Antifibrosis treatments commenced after 3 weeks of CCl_4_ injection, at which point moderate liver fibrosis had developed (Fig. 6C). The IC, ICM, ICMA, or ICMA@CZ with an identical 4-OI dosage of 2.5 mg kg^−1^ was intravenously injected into the mouse model separately every 3 days. Alanine aminotransferase (ALT) and aspartate transaminase (AST) are two major liver enzymes released into the bloodstream during liver injury (*40*). Normalization of serum levels for both enzymes was achieved solely through the ICMA@CZ treatment (Fig. 6D). Hematoxylin and eosin (H&E) staining was performed to assess the extent of liver damage (Fig. 6E). Liver tissue samples from the control group exhibited normal lobular architecture, characterized by radiating hepatic cords leading to the central veins, whereas the model group displayed fatty degeneration, ballooning of hepatocytes and necrosis. In contrast, the ICM and ICMA treatment remarkably ameliorated the adipose degeneration of hepatocytes compared to the model group. Notably, after treatment with ICMA@CZ, liver tissue samples closely resembled those of healthy liver. Liver fibrosis in mice that received CCl_4_ was confirmed through several lines of evidence. First, the accumulation of an ECM, particularly collagen, was clearly revealed by Sirius Red staining. As shown in Fig. 6 (E and F), collagen staining was scarcely observed in the control liver samples except in the areas surrounding small central venous walls. In mice treated with CCl_4_ for 5 weeks, collagen was observed extending from portal area to lobular. In the ICMA@CZ treatment group, collagen levels decreased markedly (seven folds) to the levels comparable to the control group. Meanwhile, both the ICM and ICMA groups also showed a marked reduction in collagen, whereas minimal changes were observed in the IC treatment group, further demonstrating the homologous targeting of MMs. The collagen (a major ECM protein) deposition was also significantly down-regulated compared to the PBS or IC-treated groups (Fig. 6G). In addition, the stiffness change of liver tissue was assessed using a Chiaro Nanoindenter. As shown in Fig. 6H, the stiffness of liver tissue in the ICMA@CZ treatment group was significantly lower than that in PBS group, approaching the hardness of normal tissue. It was preliminarily proved that ICMA@CZ had the ability to degrade collagen and remodel a matrix mechanical microenvironment, thereby efficiently reversing liver fibrosis.

**Fig. 7.**
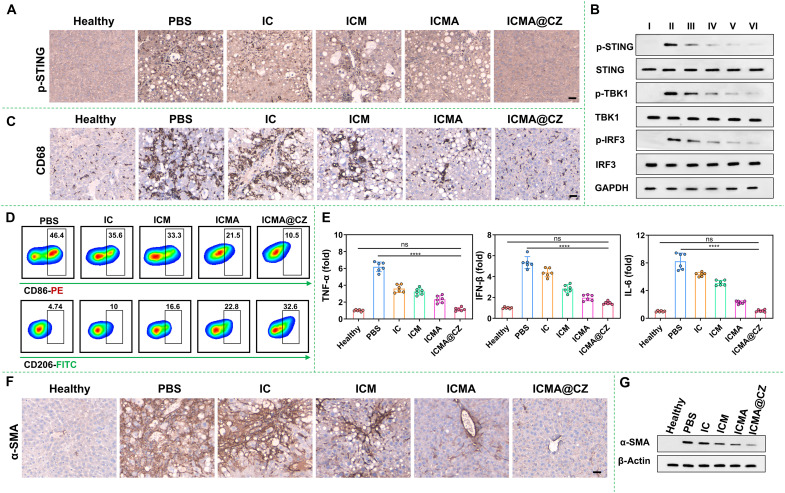
In vivo ECM-cell-ECM closed-loop reversal of liver fibrosis. (**A**) Immunofluorescence staining of p-STING in livers from the PBS, IC, ICM, ICMA, and ICMA@CZ treatment groups. Scale bar, 100 μm. (**B**) Representative Western blotting analysis of STING, TBK1, and IRF3 and phosphorylation of related proteins, which were extracted from livers after different treatments. I: healthy; II: PBS; III: IC; IV: ICM; V: ICMA; VI: ICMA@CZ. (**C**) Immunofluorescence staining of CD68 in livers from the PBS, IC, ICM, ICMA, and ICMA@CZ treatment groups. Scale bar, 100 μm. (**D**) Representative flow cytometry plots of macrophage polarization (CD86^+^ and CD206^+^ cells). (**E**) Relative mRNA expression of TNF-α, IFN-β, and IL-6 from the PBS, IC, ICM, ICMA, and ICMA@CZ treatment groups (*n* = 6). Data were analyzed using the Student’s *t* test (****P < 0.0001 versus PBS group). (**F**) Immunofluorescence staining of α-SMA in livers from the PBS, IC, ICM, ICMA, and ICMA@CZ treatment groups. Scale bar, 100 μm. (**G**) Western blot analysis of α-SMA expression after PBS, IC, ICM, ICMA, and ICMA@CZ treatment in fibrotic livers. Healthy mice used as control.

However, it was evident that the hardness of liver tissue in the ICMA treatment group was also significantly reduced compared to that in the PBS group, approaching the mechanical strength of normal liver tissue. This raises the question: What is the reason? Considering the facts that the matrix mechanics led to the highest phosphorylation of TBK1 and IRF3 (p-TBK1 and p-IRF3) that were related to the STING pathway, there was an elevation in the secretion of IL-6, TNF-α, and IFN-β, which can induce the activation of HSCs (Fig. 2). This inspired us to investigate the inhibitory effect of the ICMA on the activation of the STING pathway in vivo. As shown in Fig. 7A, p-STING (an indicator of STING pathway activation) was evaluated by immunohistochemical staining. Comparing with the healthy group, the model group exhibited a substantial increase in p-STING–positive cells in fibrotic areas and vessel walls in liver sections. In contrast, the ICMA@CZ and ICMA treatment groups demonstrated a reduced number of p-STING–positive cells compared to the model group. To quantitatively evaluate the progression of fibrosis, the percentage of p-STING–positive cells were calculated by ImageJ. As shown in fig. S16, the percentage of p-STING–positive cells increased approximately 10-fold in the 5-week CCl_4_-treated mouse model compared to the control group. Following treatment with ICMA, the percentage of p-STING–positive cells decreased by more than half, and with ICMA@CZ treatment, the percentage of p-STING–positive cells nearly returned to normal levels. In addition, the expression of p-STING, p-TBK1, and p-IRF3 was further confirmed by Western blot analysis (Fig. 7B). This analysis also revealed that the treatment with ICMA and ICMA@CZ markedly inhibited the expression of p-STING, p-TBK1, and p-IRF3. Beyond that, notable changes in p-STING, p-TBK1, and p-IRF3 were observed in the IC and ICM treatment groups (Fig. 7, A and B), indicating that the inhibition of the STING pathway was primarily mediated by 4-OI. The ability of 4-OI to inhibit the STING pathway was substantially enhanced because of the homologous targeting of macrophage cell membranes and the ECM degradation ability of CZ.

Furthermore, CD68, an activation marker of macrophage, was evaluated through immunohistochemical staining (Fig. 7C). The results showed that the number of CD68-positive cells was markedly higher in the model group than that in the healthy group. After treatment with ICMA and ICMA@CZ, a remarkable decrease in CD68-positive cells was observed, particularly treatment with ICMA@CZ, the CD68-positive cell level approached normalcy. Quantitative analysis further revealed that under treatment with ICMA and ICMA@CZ formulation, the CD68-positive cells declined by approximately four folds compared to the PBS-treated group. The reduction of CD68-positive cells in the group of ICMA@CZ was notably greater than that in the ICMA group (Fig. 4, G and H), attributable to the capacity of zinc ions to regulate the macrophage polarization (*41*). In contrast, minimal change in CD68-positive cells was noted in the IC and ICM treatment groups, indicating that the polarization of macrophages was mainly mediated by zinc ions (fig. S17). At the same time, flow cytometry was used to further assess the macrophage polarization capacity of ICMA@CZ. The results demonstrated that ICMA@CZ exhibited a superior ability to diminish the proportion of CD86^+^ cells (indicative of the M1 macrophage, decreasing from 46.4 to 10.5%) and concurrently eliciting the highest proportion of CD206^+^ macrophages (representing the M2 macrophage, reaching 25.3%). In the group of IC and ICM, a relative decrease in CD86^+^ cells and an increase of CD206^+^ macrophages were also observed (Fig. 7D). This finding aligns with the activation of STING pathway (Fig. 7, A and B), indicating that the inhibition of STING pathway activation can reduce the polarization of macrophages. In other words, activation of the STING pathway markedly induced macrophage polarization toward the M1 type. In addition, consistent with the in vitro experiments, treatment with ICMA noticeably reversed the polarization of macrophages from the M1 type to M2 type. This effect may be attributed to the anti-inflammatory properties of AP (*28*, *42*), which effectively enhanced the endocytosis of the nanocarriers and facilitated the reversal of polarization (Fig. 3F). Furthermore, comparing with PBS-treated livers, a substantial reduction of profibrogenic factors was observed, including TNF-α (five folds), IFN-β (five folds), IL-6 (eight folds), and transforming growth factor–β (TGF-β; seven folds) in the livers of mice treated with ICMA@CZ (Fig. 7E and fig. S18). These results further proved that STING activation induced macrophage polarization and promoted the release of inflammatory factors.

As demonstrated in our previous work (*10*), undesirable cross-talk existed between macrophages and HSCs. Subsequently, α-SMA, an indicator of the activation of HSCs, was evaluated by immunohistochemical staining (Fig. 7F) and Western blot analysis (Fig. 7G). The percentage of α-SMA–positive cells increased by about 20 folds in the 5-week CCl_4_-treated mouse model than that in the healthy group. After treatment with ICMA@CZ, the percentage of α-SMA–positive cells nearly reversed almost to the normal levels. Beyond that, minimal changes in α-SMA were found in the IC and ICM treatment groups (fig. S19). These findings were consistent with collagen and the matrix mechanics (Fig. 6, G and H). It was clearly indicated that the carrier-free nanosystem containing matrix metalloproteinases and STING inhibitors can efficiently target macrophages and degrade the ECM obviously, thus improving liver fibrosis in liver injury. No histological abnormities were induced in major organs (fig. S20), nor were any sremarkable changes in body weight caused (fig. S21). In addition, serum levels for both AST and ALT in AALD mice treated with ICMA@CZ remained normal (Fig. 6D). Even after healthy mice were treated with these nanosystems, there were no changes in either blood biochemistry markers (ALT and AST) or immunological markers (IL-6 and TNF-α) (fig. S22), suggesting negligible systemic toxicity and low immunogenicity. In the AALD group, the liver index increased significantly; however, after treatment with ICMA@CZ, a noticeable reduction in the liver index was observed (fig. S23). Collectively, matrix degradation can effectively promote the deep penetration of nanoparticles, remodel matrix mechanics, regulate macrophage polarization, and blocking the activation of the STING pathway in macrophages can effectively reverse the activation of HSCs and further remodel the matrix mechanics, thus achieving the ECM-cell-ECM closed-loop reversal of liver fibrosis.

## DISCUSSION

There are substantial research opportunities in reversing liver fibrosis, particularly in exploring the relationship between mechanical forces and the development of this condition. Studies have indicated that macrophage activity is influenced by mechanical properties, which can lead to aging or fibrosis (*18*). However, the precise response of macrophages to the mechanical properties of the ECM remains unclear. Here, we have demonstrated the existence of an ECM-cell-ECM loop, where enhanced ECM stiffness induces macrophage polarization through the activation of the STING pathway, subsequently activating HSCs to further reinforce the mechanical properties of the matrix, ultimately exacerbating liver fibrosis. On the basis of this, we designed a matrix mechanical remodeled carrier-free nanosystem (ICMA@CZ). This nanosystem facilitated the loading of matrix metalloproteinase (CZ) onto its surface, allowing for ECM degradation to enhance the deep penetration of ICMA. In addition, Zn^2+^ can be released to modify macrophage polarization. Then, the ICMA nanosystem was engineered to target predominant macrophages in the liver by using MM coatings, enabling the targeted release of 4-OI to inhibit STING pathway activation in the macrophages. This approach collectively regulated the polarization state of macrophages, reduced the release of inflammatory factors, inhibited HSC activation, and decreased ECM production, consequently leading to a down-regulation of matrix mechanical strength and interruption of the ECM-cell-ECM cycle. As a result, a closed-loop reversal of liver fibrosis was achieved. In a mouse model of AALD with middle liver fibrosis, the ICMA@CZ nanosystem degraded the ECM and regulated the macrophage polarization by blocking the STING pathway. Furthermore, the matrix mechanical strength was down-regulated by inhibiting the activation of HSCs, thus leading to an ECM-cell-ECM closed-loop therapeutic effect in AALD with middle liver fibrosis–bearing mice.

Although the literature indicated that the macrophages can respond to matrix mechanical changes to activate HSCs, thereby promoting the development of liver fibrosis (*43*), it also suggested that regulating matrix mechanical forces may serve as an innovative target for reversing liver fibrosis. However, there has been no report on how to regulate mechanical forces to reverse liver fibrosis. In this study, the construction of carrier-free nanocarriers notably enhanced drug loading efficiency, as well as avoiding the risk of systemic toxicity associated with the introduction of exogenous materials (*44*). Simultaneously, the metal ion–based loading of proteases effectively addressed the challenge of insufficient enzyme activity in the lesion area due to the depletion of metal ions (such as zinc ions) (*45*, *46*). Also, the presence of these metal ions further facilitated the polarization regulation of macrophages, maximizing material utilization while ensuring the safety of the nanosystem. In addition, the nanoassembly technology allowed for the easy incorporation of other functional modules as needed, laying a theoretical foundation for the development of customized material preparations.

All in all, this study establishes a mechanism through which macrophages respond to the mechanical properties of the ECM to induce liver fibrosis. It also highlights the considerable potential of a matrix mechanical remodeled, carrier-free nanosystem for programmatically disrupting the ECM-cell-ECM cycle, thereby achieving a closed-loop reversal of liver fibrosis. Given the ease metal ion–based loading of proteases and their high enzymatic activity retention, along with the role of metal ions in facilitating liver fibrosis treatment, this study opens avenues for the efficient loading of proteases and the development of precise diagnostic and therapeutic nanosystems based on in-depth understanding of the mechanism underlying the influence of matrix mechanics on the development of liver fibrosis.

## MATERIALS AND METHODS

### Materials

4-OI was purchased from Macklin (Shanghai, China). AP, ICG, zinc sulfate heptahydrate, and methacrylate anhydride (MA) were purchased from Heowns (Tianjin, China). Collagenase I, LPSs, RPMI 1640 medium, and MTT were obtained from Solarbio life Sciences (Beijing, China). Dulbecco’s modified Eagle’s medium (DMEM) and FBS were purchased from Gibco. Phenylmethylsulfonyl fluoride (PMSF) was purchased from Thermo Fisher Scientific. Hoechst 33342 was obtained from Beyotime Institute of Biotechnology (Shanghai, China). All reagents were used without further purification or modification.

### Preparation of GelMA and in vitro matrix mechanical environment

The GelMA was synthesized according to relevant literature, with minor adjustments made to specific details (*47*, *48*). First, 15 g of gelatin was dissolved in 300 ml of PBS buffer by heating to 50°C under continuous stirring until complete dissolution. Then, 6 ml of MA was added slowly, followed by vigorous stirring for 3 hours to ensure full reaction. Unreacted MA was subsequently removed via dialysis using a dialysis bag (7 to 14 kDa) for 3 days. The resultant reaction products were collected and freeze-dried for further use.

To mimic the in vitro matrix mechanical environment, the GelMA hydrogels with varying stiffness levels were prepared by adjusting the concentration of GelMA. Specifically, GelMA was dissolved in a preprepared 0.25% lithium phenyl (2,4,6-trimethylbenzoyl) phosphinate solution, followed by irradiation with 405-nm blue light for 50 s to form 5, 15, and 30% GelMA hydrogels. The loss modulus and storage modulus were characterized using a rheometer (MCR302, Anton Paar, Austria). The effective surface modulus of GelMA hydrogels was determined using a Chiaro Nanoindenter (Optics11 Life, the Netherlands).

### ^1^H NMR characterization of GelMA

The chemical structure of GelMA was verified by ^1^H nuclear magnetic resonance (NMR) spectrometry (AVANCE NEO 800, Bruker, Switzerland) (fig. S2). The characteristic NMR resonances belonging to MA could be clearly detectable. The new peaks at 5.32 and 5.56 parts per million (ppm) were attributed to the double-bonded protons of MA groups. In addition, a notable reduction in the signal of free lysine at 2.90 ppm was observed. The MA grafting efficiency was calculated to be 64% by integrating the peak area of the lysine methylene of gelatin at 2.90 ppm.

### Preparation of the 4-OI loading carrier-free nanosystem (IC)

The IC carrier-free nanosystems were synthesized using a reprecipitation method (*38*). First, 100 μl of the prepared 4-OI tetrahydrofuran solution (1 mg/ml) was added slowly to 5 ml of deionized water while continuously stirring at room temperature for 15 min. Next, 200 μl of the prepared ICG solution (1 mg/ml) was added dropwise into the mixture, followed by overnight stirring in the dark to facilitate the formation of IC nanoparticle. The resulting nanoparticles were filtered through 800-, 450-, and 220-nm water-phase filters before use.

### Extraction of RAW 264.7 cell membranes (MMs)

According to the manufacturer’s protocol (Beyotime Biotechnology), the macrophage cell membranes were extracted using the Membrane and Cytosol Protein Extraction Kit. The cells were collected and centrifuged at 1000*g* for 5 min. Following that, the cells were resuspended in precooled PBS and centrifuged for 5 min at the rapid of 600*g*, and then, the supernatant was discarded. Subsequently, 1 ml of membrane protein extraction reagent A (supplemented with PMSF) was added, and the cells were resuspended and incubated on ice for 10 to 15 min. The cells were then lysed through repeated freeze-thaw cycles (four to five times) at −80°C, followed by centrifugation at 700*g* for 10 min at 4°C. The supernatant was collected and subjected to further centrifugation for 30 min at 14,000*g* to isolate the RAW 264.7 cell membranes (MMs). The cell membranes were stored at −80°C. Before use, the lyophilized membranes were rehydrated in deionized water.

### Fabrication and characterization of ICM and ICMA

The ICM was synthesized on the basis of our previous work (*26*). Briefly, the IC nanosystem prepared above was mixed with MM dispersion in a defined ratio under vortex stirring and incubated at room temperature for 10 min. Subsequently, it was extruded for at least 3 cycles through a series of water-phase filters with decreasing pore sizes of 800, 450, and 220 nm. ICM nanoparticles were prepared with different mass ratios of membrane to IC, ranging from 2 to 5. To synthesize the ICMA, the prepared AP dimethyl sulfoxide solution (5 mg/ml) with various volumes was added into the ICM solution and incubated at room temperature for 15 min. Then, the hydrodynamic size and zeta potential of ICM and ICMA were determined using DLS (Malvern Instrument, Zetasizer Nano ZS) at 25°C. The data were shown as mean ± SD based on three independent measurements.

### Fabrication and characterization of matrix mechanical remodeled nanosystem (ICMA@CZ)

The ICMA@CZ nanosystem was fabricated by adding different volumes of metal ion–complexed collagenase (CZ) solution into the ICMA solution. Briefly, 10 mg of collagenase I was dissolved into 2 ml of deionized water. The CZ nanoparticles were synthesized by slowly adding 80 μl of 0.1 M ZnSO_4_ solution to the above solution while stirring, followed by adjusting the pH to 10. The resulting solution was stirred at room temperature for another 3 hours. Subsequently, the ICMA@CZ nanosystem was synthesized by mixing varying volumes of CZ solution (5 mg/ml) into the ICMA solution under vortex stirring and then incubated at room temperature for 15 min. The average particle size and zeta potential of ICMA@CZ were determined using DLS as described above. The drug loading efficiency of 4-OI was quantified using HPLC (Waters Corporation, USA) at a wavelength of 210 nm, and the encapsulation efficiency (EE) was calculated according to the following equation

$$\begin{array}{c} \text{EE}\;\left(\%\right)=\left(\text{mass of drug encapsulated in nanoparticles}\right)/\\ \left(\text{mass of drug added}\right)\times 100 \end{array}$$

The morphologies of nanoparticles were characterized using a transmission electron microscope (JEOL JEM-2100F, Japan) with an accelerating voltage of 80 kV. Before visualization, the nanoparticles were dispersed onto a carbon-coated copper grid. The stability of the nanoparticles was assessed by evaluating serum adsorption behavior using a microplate reader (Infinite M200 PRO, Tecan, Switzerland).

### Cell culture conditions

RAW 264.7 cells were cultured in DMEM medium supplemented with 10% FBS at 37°C in a humidified incubator with 5% CO_2_. Rat HSCs (HSC-T6) and human HSCs (LX-2) were cultured under the same condition. Mouse KCs were cultured in RPMI 1640 supplemented with 10% FBS in a humidified incubator with 5% CO_2_ at 37°C.

### Response of macrophages to changes in matrix stiffness

To investigate the response of macrophages to changes in matrix stiffness, the variation in Ca^2+^ concentration and mitochondrial membrane potential in macrophages under different matrix mechanical microenvironments was measured. For the determination of Ca^2+^ concentration, RAW 264.7 cells were seeded onto hydrogel surfaces with varying stiffness at a density of 1 × 10^5^ cells per well and incubated at 37°C for 24 hours. Subsequently, the cells were harvested and stained with a mitochondrial Ca^2+^ indicator (Rhod-2 AM) (MedChemExpress) for 30 min at room temperature. To analyze the changes in mitochondrial membrane potential in RAW 264.7 cells, the collected RAW 264.7 cells from different mechanical microenvironments were stained with JC-1 (MedChemExpress) for 30 min. Following that, the cells were collected and washed twice with PBS. All the fluorescence was evaluated using an inverted microscope (Invitrogen, EVOS M5000, USA). ImageJ was used for semiquantitative analysis.

### In vitro homotypic targeting assay

To assess the homotypic targeting ability of MM-coated nanoparticles, an inverted microscope was used to determine the cellular uptake efficiency of various nanoparticles in RAW 264.7, HSC-T6, LX-2 cells, and KCs. The cells were seeded in 24-well plates at a density of 1 × 10^4^ cells per well and cultured for 24 hours. After that, the supernatant was removed, and the cells were incubated with fresh medium containing various nanosystems (2 μg/ml) for 1 hour. Subsequently, the supernatant was discarded, and the cells were washed thrice with 500 μl of PBS. Throughout the entire process, exposure to strong light was avoided to protect the fluorescent dyes. Observations in HSC-T6 and LX-2 served as controls.

### Deep penetration ability assay

A transwell model was used to validate the capability of the nanosystem to degrade collagen caused by the mechanics of matrix and achieve deep penetration. First, RAW 264.7 cells were stimulated with LPS (100 ng/ml) or the hydrogel with different stiffness (0.1 to 5 kPa) for 24 hours. The supernatant was collected as the CM for further use. Subsequently, HSC-T6 or LX-2 cells were seeded into the upper chambers of the transwell plate at a density of 1 × 10^4^ cells per well and cultured for 24 hours. Then, the medium was replaced with either the CM described above or fresh medium and cultured for an additional 24 hours. After that, the cells were treated with/without medium containing CZ for another 2 hours. Meanwhile, RAW 264.7 cells were seeded into the lower chambers of transwell plate at the same density and cultured independently for 24 hours. Following that, the medium of HSC-T6 or LX-2 cells was replaced with fresh medium containing various nanosystems, and these cells were cocultured with the RAW 264.7 cells in the lower chambers for 2 hours. Last, the RAW 264.7 cells were washed using PBS buffer solution for three times, and the fluorescence intensity within the cells was observed using an inverted fluorescence microscope (Invitrogen, EVOS M5000, USA).

### Confocal laser scanning microscopy

To elucidate the collagen degradation ability of ICMA@CZ, HSC-T6 cells were seeded in confocal dishes or six-well plates at a density of 5 × 10^4^ cells per well and cultured for 24 hours. Subsequently, the culture medium was replaced with CM described above, and the cells were incubated for an additional 24 hours. Then, the different nanosystems were added, and the cells were further cultured for 2 hours. After that, the medium was collected, and the total collagen was measured using the Sirius Red Total Collagen Detection Assay Kit (Chondrex). The cells were washed several times with PBS solution, fixed with 4% paraformaldehyde for 15 min, blocked for 1 hour with blocking solution (5% BSA and 0.2% Triton X-100). Then, the cells were incubated at 4°C overnight with α-SMA primary antibody (1:200). Afterward, the cells were rinsed three times in PBS for 5 min each and incubated with anti-rabbit immunoglobulin G Alexa Fluor 488 secondary antibody (Thermo Fisher Scientific) at room temperature for 1 hour. Nuclei were stained with Hoechst 33342 for 15 min and visualized under a confocal laser scanning microscopy (CLSM) (Nikon A1R+, Shanghai, China; excitation filter of 488 nm and emission cutoff filter of 515 to 530 nm for green light). Initial HSC-T6 cells served as the negative control, and CM-activated HSC-T6 served as the positive control.

To explore the ability of zinc ions (Zn^2+^) to regulate the polarization of macrophages, RAW 264.7 cells were seeded in confocal dishes at a density of 5 × 10^4^ cells per well, cultured for 24 hours, and stimulated with LPS for another 24 hours. Subsequently, the medium was replaced with fresh medium containing different nanosystems, and the cells were further incubated for 2 hours. After that, the supernatant was discarded, and the cells were incubated in the medium containing phycoerythrin (PE)–conjugated anti-mouse CD86 antibody (Elabscience) and fluorescein isothiocyanate (FITC)–conjugated anti-mouse CD206 antibody (Elabscience) at 4°C for 30 min. Nuclei were stained with Hoechst 33342 at room temperature for 15 min, and then the cells were washed several times using PBS buffer solution and imaged under a CLSM (Nikon A1R+, Shanghai, China).

### Flow cytometry analysis

The cellular internalization of nanosystems in RAW 264.7 cells was quantitatively evaluated using two-color flow cytometry. Briefly, RAW 264.7 cells were seeded onto six-well plates at a density of 1 × 10^5^ cells per well and incubated for 24 hours. The supernatant was replaced with fresh medium containing varied nanosystems, and the cells were further cultured for 1 hour. Subsequently, the cells were washed three times with PBS and harvested by trypsinization. The cells were then resuspended in 500 μl of PBS, and fluorescence intensity was detected using flow cytometry (Beckman, CytoFLEX, USA). HSC-T6 cells, LX-2 cells, and KCs treated in the same manner served as controls, while blank cells were used as a negative control.

For macrophage polarization mediated by Zn^2+^, RAW 264.7 cells were stimulated with LPS (100 ng/ml) for 24 hours. Following that, the cells were treated with ICMA or ICMA@CZ for 2 hours. After treatment, the cells were washed with PBS and collected. For tissue samples, liver tissues from different groups of mice were homogenized in PBS using a glass grinder and filtered through a cell filter to prepare a single-cell suspension. The cells were then incubated with PE-conjugated anti-mouse CD86 antibody (Elabscience) and FITC-conjugated anti-mouse CD206 antibody (Elabscience) for 30 min. Last, the cells were washed with PBS and resuspended in 500 μl of PBS, and the fluorescence intensity was detected using flow cytometry (Beckman, CytoFLEX, USA).

### Blocking of STING pathway assay

To investigate the ability of nanosystems to block of STING pathway in macrophages, RAW 264.7 cells were cultured in 24-well plates at a density of 1 × 10^4^ cells per well for 24 hours. The medium was then replaced with fresh medium containing various nanosystems, followed by incubation for 1 hour. Afterward, the medium was removed, and the cells were stimulated with LPS or GelMA hydrogels with different stiffness for 24 hours. Cells stimulated with an agonist of the cGAS-STING pathway (DMXAA) served as a positive control. Last, supernatants from the DMXAA-stimulated cells were collected and used to culture HSC-T6 cells, and the cytokine concentrations were measured using an enzyme-linked immunosorbent assay (ELISA) kit (BioLegend).

To demonstrate the existence of ECM-cell-ECM cycle based on the STING pathway in macrophage, RAW 264.7 cells were seeded onto six-well plates at a density of 1 × 10^5^ cells per well and cultured for 24 hours. The cells were then treated with fresh medium containing various nanosystems for 1 hour. Afterward, the cells were harvested and reseeded on the surface of prepared GelMA hydrogels with different stiffness. After incubation for 24 hours, the supernatants were collected and used as CM to culture HSC-T6 cells for 24 hours. Cytokine concentrations were quantified using an ELISA kit (BioLegend), and the total collagen content was quantified using the Sirius Red Total Collagen Detection Assay Kit (Chondrex). The effective surface modulus of HSC-T6 before and after treatment was assessed using a Chiaro Nanoindenter (Optics11 Life, the Netherlands).

### Biodistribution of ICMA@CZ nanoparticles in mice with liver fibrosis

All animal experiment protocols were approved by the Animal Care and Use Committee (Institute of Radiation Medicine, Chinese Academy of Medical Sciences and Peking Union Medical College, IRM/2-IACUC-2504-001). Female C57BL/6J mice (7 weeks old, 20 g body weight) purchased from the Vital River Laboratory Animal Technology Co. Ltd. (Beijing, China) were fed with 1% ethanol diet for 2 days. On day 3, the diet was changed to 2% ethanol for the remainder of the experiment. Starting from day 5, the mice were intraperitoneally injected with 10% CCl_4_ olive oil (1 ml/kg, twice a week) for 5 weeks to induce fatty liver with mild fibrosis. On day 35, the mice received an intravenous injection of free ICG, IC, ICMA, and ICMA@CZ. After 12 or 24 hours, the mice were euthanized, and ex vivo fluorescence imaging of the main organs was performed using a multimodal animal imaging system (Biolight Biotechnology, AniView600, Guangzhou, China).

### In vivo liver fibrosis reversal

C57BL/6J mice were randomly assigned to six groups: healthy, PBS, IC, ICM, ICMA, and ICMA@CZ. Treatments were administered every 3 days, while healthy and untreated mice served as controls. Body weights were monitored throughout the experiment. Whole blood was collected on days 17 and 35. On day 36, all mice were euthanized, and the organs were harvested for histological and biomedical analyses. A portion of liver tissues was collected for Western blotting, flow cytometry, and quantitative reverse transcription polymerase chain reaction (qRT-PCR) analysis, and the other tissues were fixed with 4% paraformaldehyde, embedded in paraffin, sectioned, and stained with H&E. Sirius Red and immunohistochemical staining (α-SMA, CD68, and p-STING) were performed on liver tissues. The effective surface modulus of fibrotic livers before and after treatment was assessed using a Chiaro Nanoindenter (Optics11 Life, the Netherlands).

The mRNA levels of TNF-α, IL-6, IFN-β, COL-I, and TGF-β in the liver were quantified by qRT-PCR. Total RNA was extracted from liver lysates using TRIzol reagent according to the manufacturer’s instructions (Hippobio, China), converted into cDNA using a cDNA transcription kit (Hippobio, China). PCR was conducted using HPOGreen qPCR Master Mix (Hippobio, China), and relative gene expression was calculated using the ΔΔCt method with glyceraldehyde phosphate dehydrogenase (GAPDH) as the internal reference. Primer sequences were shown in table S1.

### Blood chemical analysis

Whole blood cell samples were centrifuged at 6000 rpm for 15 min at 4°C to obtain serum. The levels of AST and ALT in the serum were measured using AST and ALT assay kits according to the manufacturer’s protocols (Solarbio, China).

### Statistical analysis

All experiments were repeated at least three times and presented as the means ± SD. The statistical analysis was performed using GraphPad Prism 10.1.2 software. Statistical differences were calculated with the two-tailed Student’s *t* test; *P < 0.05, **P < 0.01, ***P < 0.001, and ****P < 0.0001.
