# Supplementary material for: Matrix mechanical remodeled carrier-free nanosystem for programmable closed-loop reversal of liver fibrosis via STING alkylation
Source: Sci Adv. 2025 Nov 19;11(47):eadz4126. doi: 10.1126/sciadv.adz4126 (PMC12629182; doi:10.1126/sciadv.adz4126)
Supplement: Supplementary file 1 — Figs. S1 to S23 Table S1 [file sciadv.adz4126_sm.pdf]

Science Advances
AAAS

Supplementary Materials for

Matrix mechanical remodeled carrier-free nanosystem for programmable closed-loop reversal of liver fibrosis via STING alkylation

Hongyun Han *et al.*

Corresponding author: Huizhen Jia, huizhen.jia@tju.edu.cn

Sci. Adv. **11**, eadz4126 (2025)
DOI: 10.1126/sciadv.adz4126

This PDF file includes:

Figs. S1 to S23
Table S1

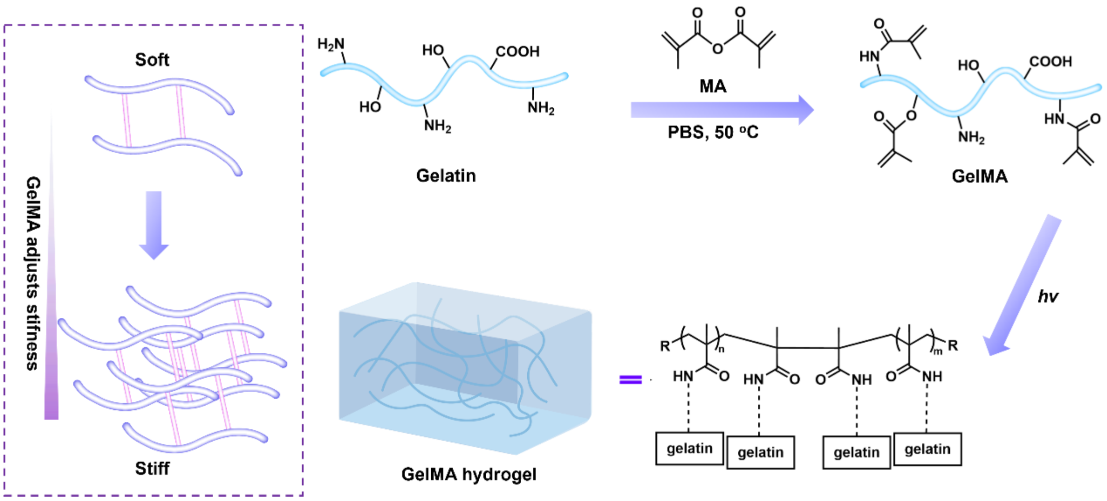

Fig. S1. Synthesis of GelMA hydrogel.

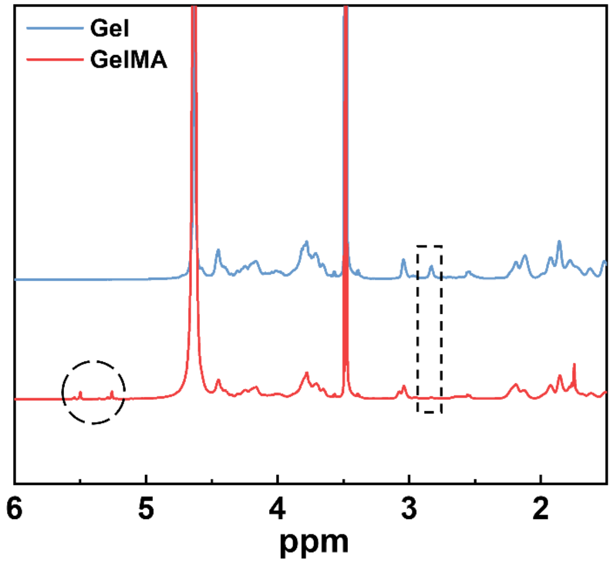

Fig. S2. ^{1}H NMR spectra of GelMA and Gel in D_2O.

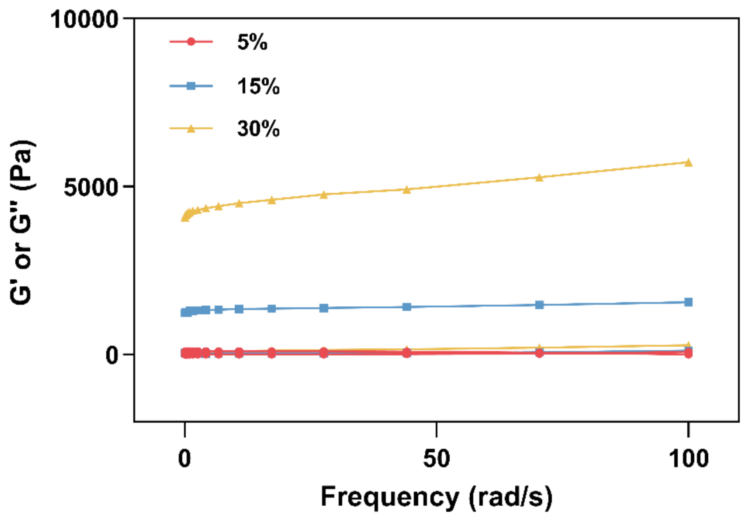

Fig. S3. Representative oscillatory frequency sweep (0.1-100 rad/s) of GelMA hydrogels of different concentrations.

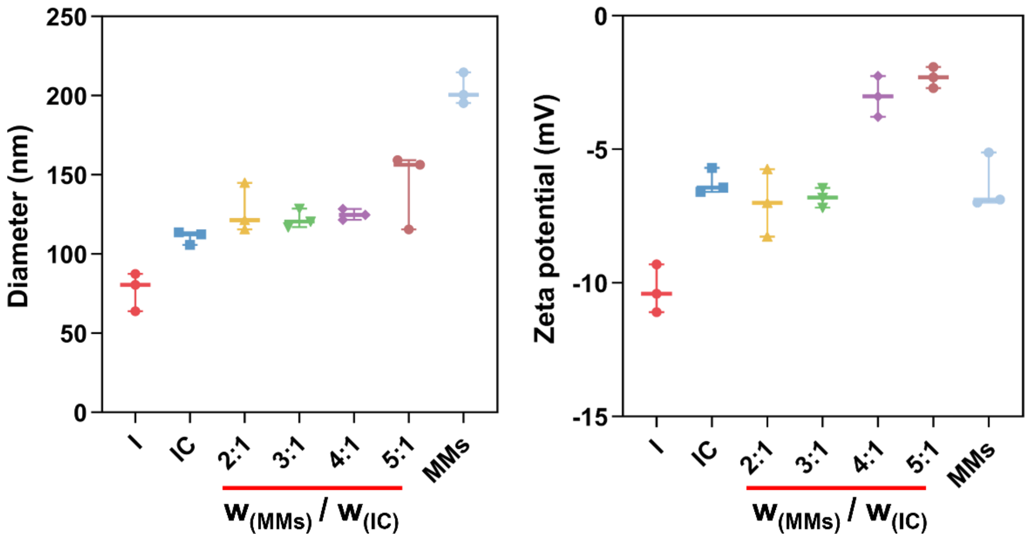

Fig. S4. Particle size and zeta potential of ICM at different weight ratios of MMs *versus* IC (the size and zeta potential of MMs were used as a control).

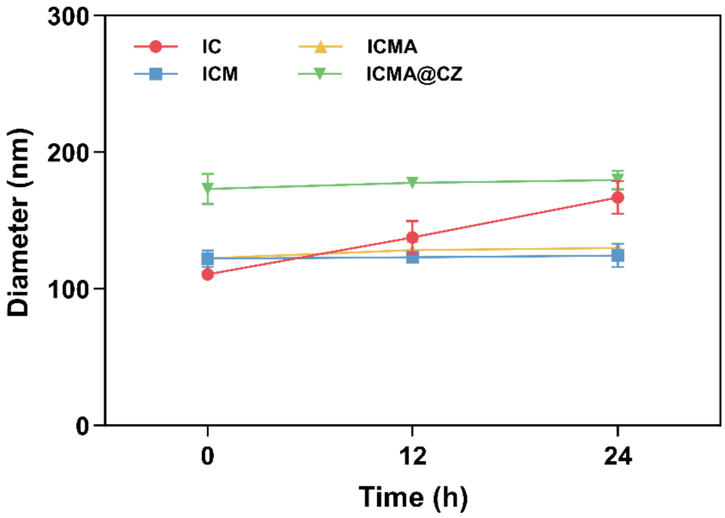

Fig. S5. Variation in the hydrodynamic diameters of IC, ICM, ICMA and ICMA@CZ as a function of time in DI water.

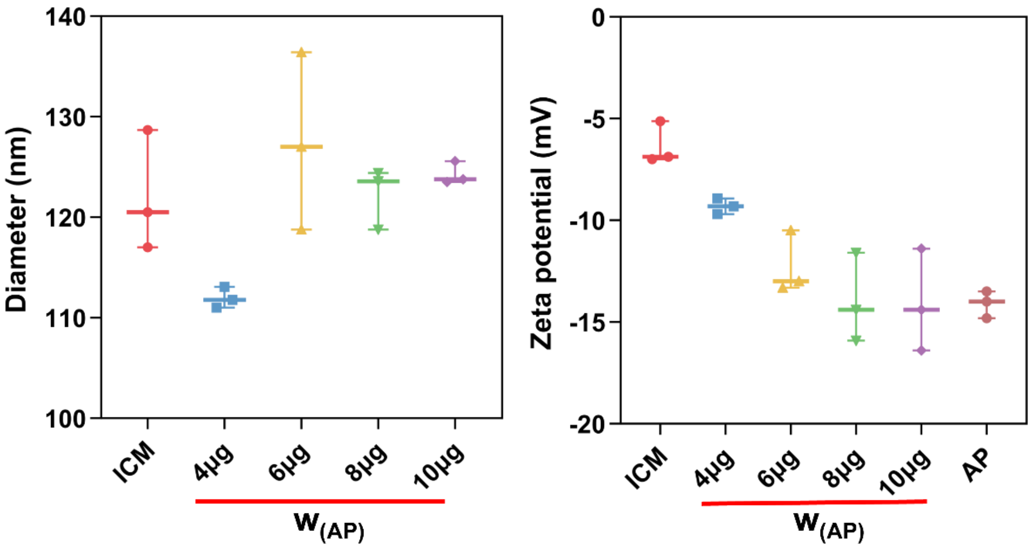

Fig. S6. Particle size and zeta potential changes of ICM after addition of AP with different concentrations.

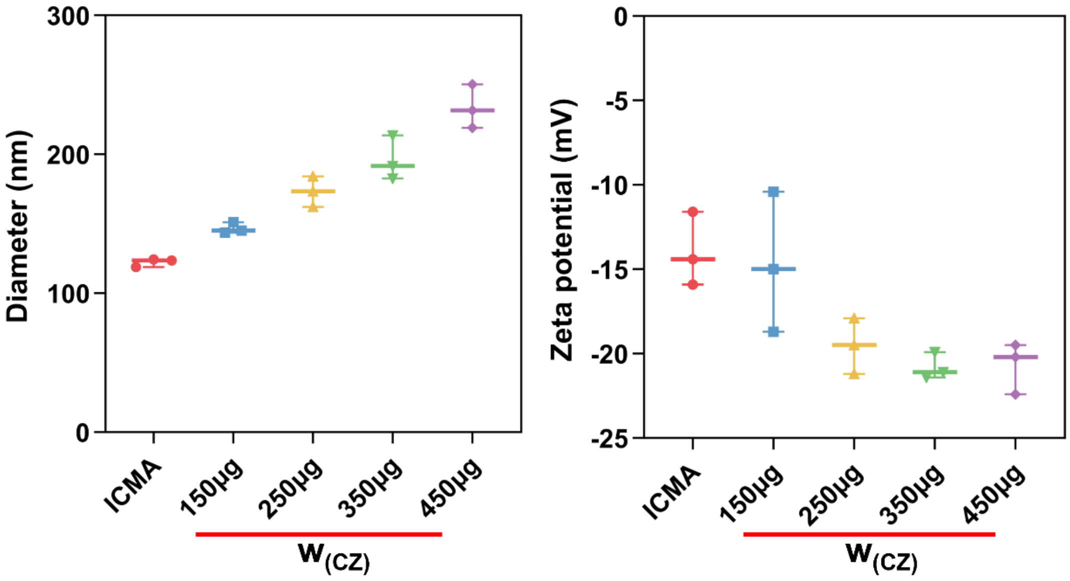

Fig. S7. Particle size and zeta potential changes of ICMA after addition of CZ with different concentrations.

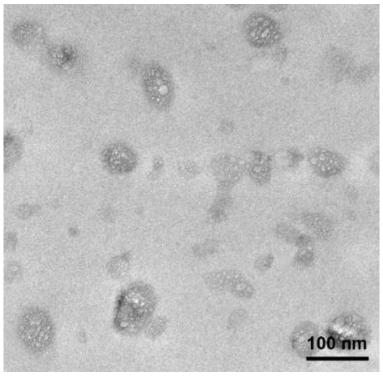

Fig. S8. TEM image of CZ.

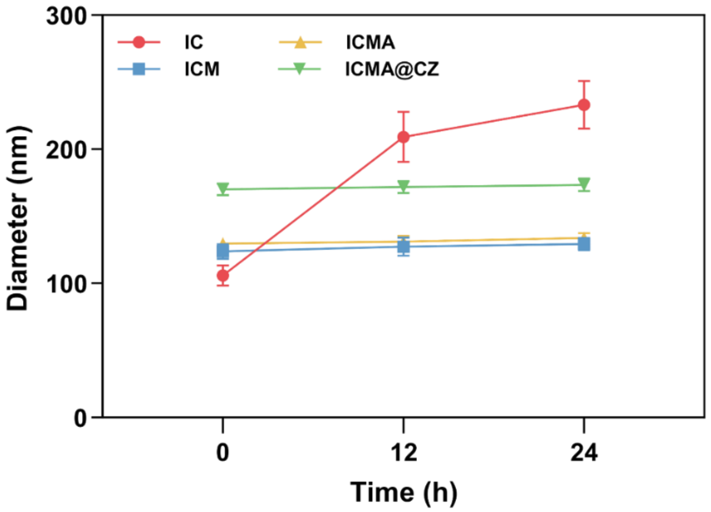

Fig. S9. Variation in the hydrodynamic diameters of IC, ICM, ICMA and ICMA@CZ over time in PBS containing 10% FBS.

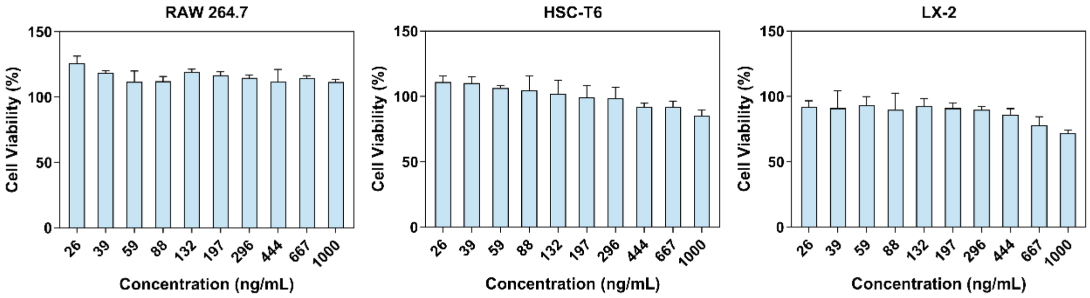

Fig. S10. *In vitro* cytotoxicity of ICMA@CZ at different concentrations on RAW 264.7, HSC-T6 and LX-2 cells after 24 hours incubation.

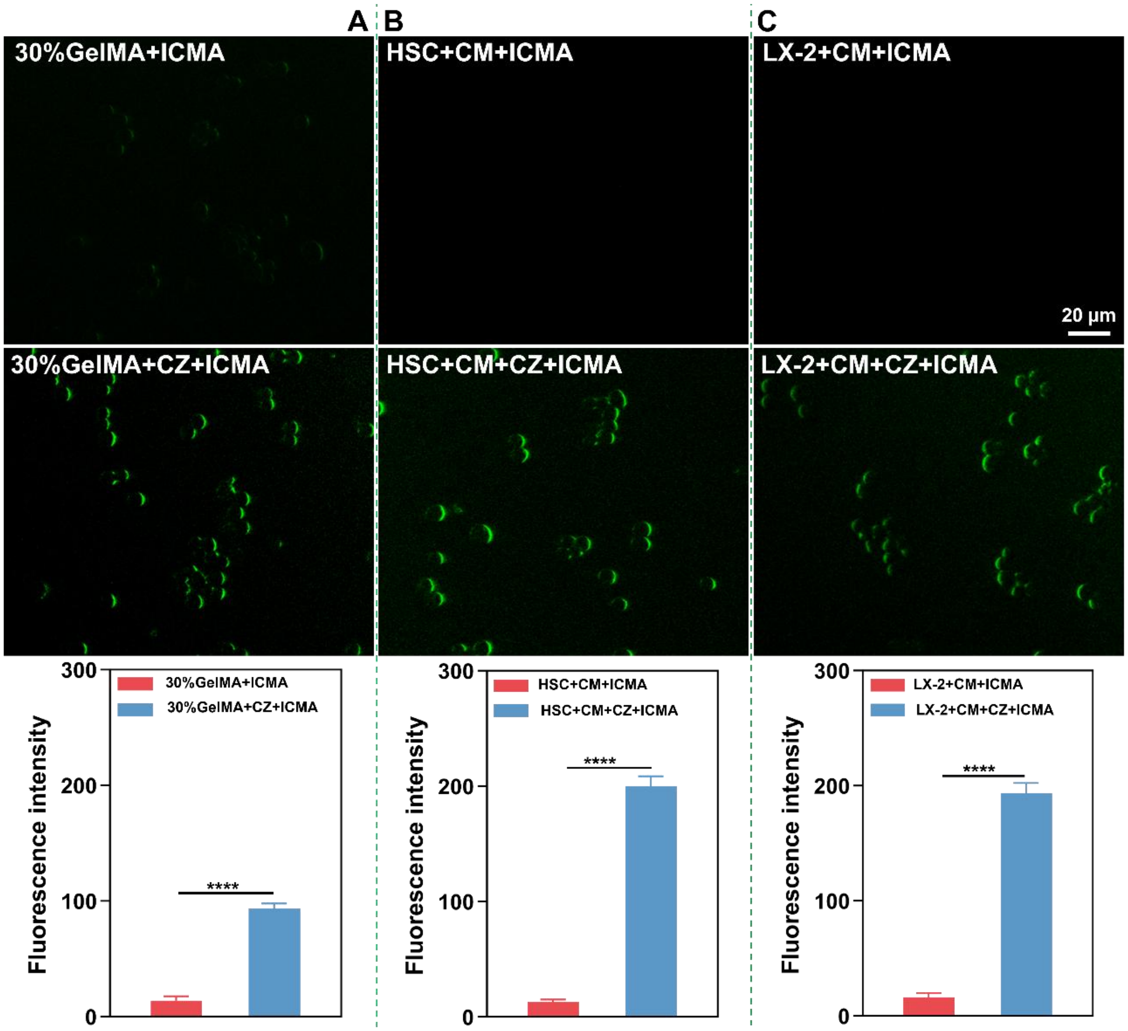

Fig. S11. Matrix mechanical remodeling through ECM degradation mediated by free CZ. (**A**) Microphotographs and mean fluorescence intensity (MFI) of RAW 264.7 with adding of ICMA for 2 h after co-cultured with HSC-T6 cells using transwell plate. The HSC-T6 cells were treated with the conditioned medium (CM) from RAW 264.7 cells stimulated by hydrogel with 5 kPa for 24 h and subsequently treated with/without CZ for another 2 h before adding of ICMA. Scale bars: 20 μm; (**B, C**) Microphotographs and MFI of RAW 264.7 with adding of ICMA for 2 h following co-cultured with HSC-T6 and LX-2 cells. The HSC-T6 and LX-2 cells were treated with the CM from RAW 264.7 cells with stimulation of LPS for 24 h and then treated with/without CZ for another 2 h before adding of ICMA. Scale bars: 20 μm.

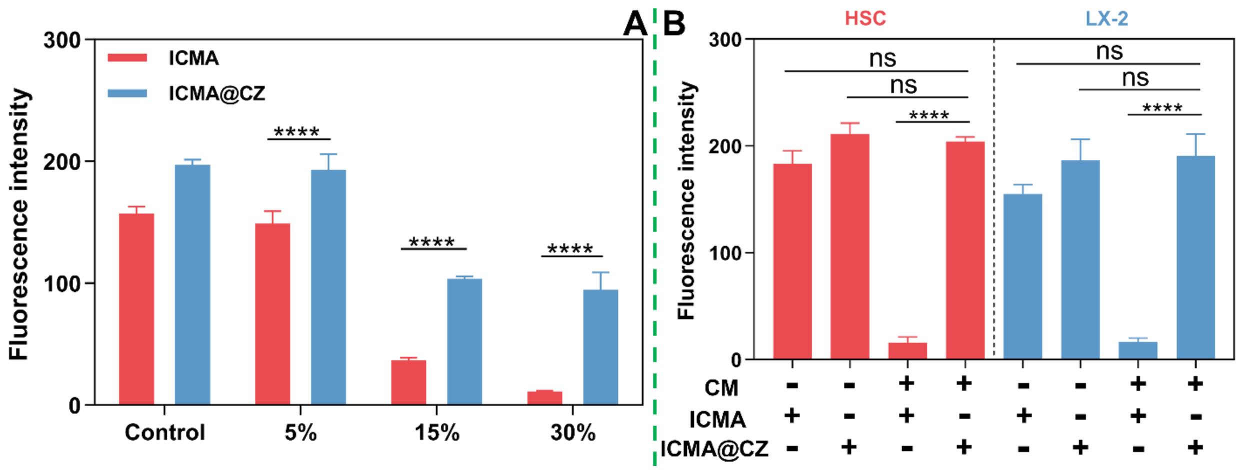

Fig. S12. Quantitative analysis of the data from Fig. 4B and D. (**A**) Mean fluorescence intensity (MFI) of intracellular ICMA and ICMA@CZ from Fig. 4B were analyzed using Image J software; (**B**) Mean fluorescence intensity (MFI) of ICMA and ICMA@CZ-positive cells from Fig. 4D were analyzed using Image J software.

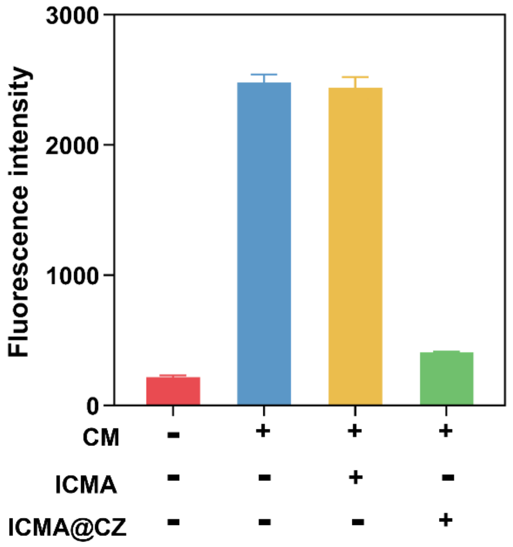

Fig. S13. Mean fluorescence intensity (MFI) of α-SMA-positive cells from Fig. 4E were analyzed using Image J software.

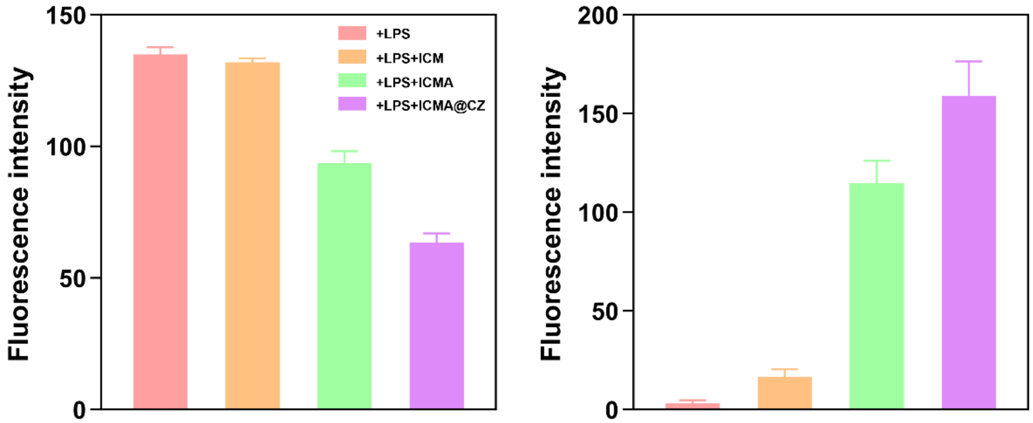

Fig. S14. Fluorescence of CD86 (marker for M1 phenotypes) and CD206 (marker for M2 phenotypes) in the polarization of RAW 264.7 macrophages was analyzed using Image J software.

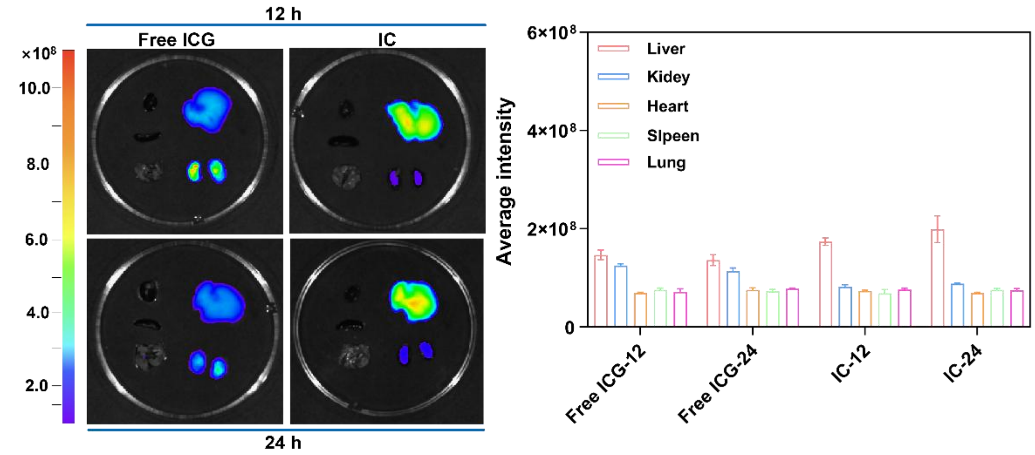

Fig. S15. *Ex vivo* fluorescence imaging and quantification of fluorescence distribution in major organs after intravenously injection of free ICG and IC for 12 h and 24 h (n = 3, mean ± SD).

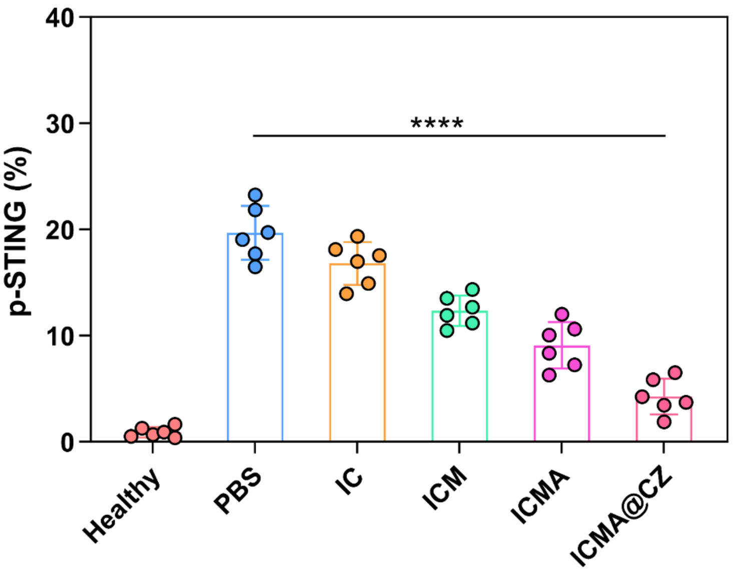

Fig. S16. Quantification analysis of p-STING in CCl_4-induced fibrotic livers from the PBS, IC, ICM, ICMA or ICMA@CZ treatment groups was performed using Image J software (n = 6). Data were analyzed using Student's t-test (****p < 0.0001 *versus* PBS group).

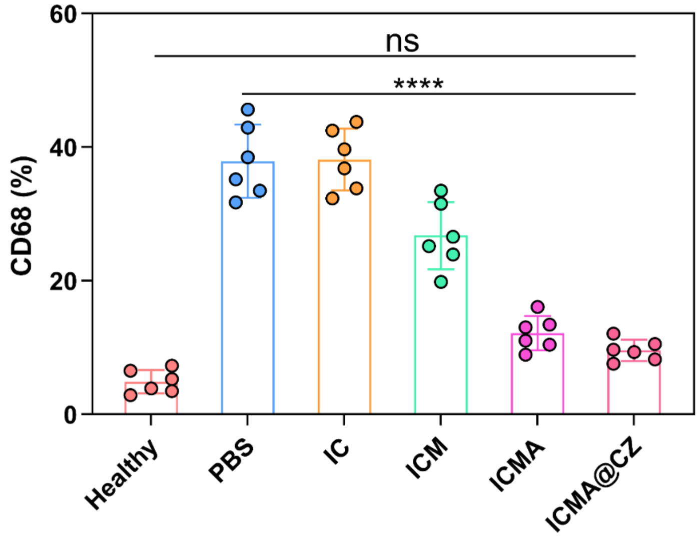

Fig. S17. Quantification analysis of CD68 in CCl_4-induced fibrotic livers from the PBS, IC, ICM, ICMA or ICMA@CZ treatment groups was performed using Image J software (n = 6). Data were analyzed using Student's t-test (****p < 0.0001 *versus* PBS group).

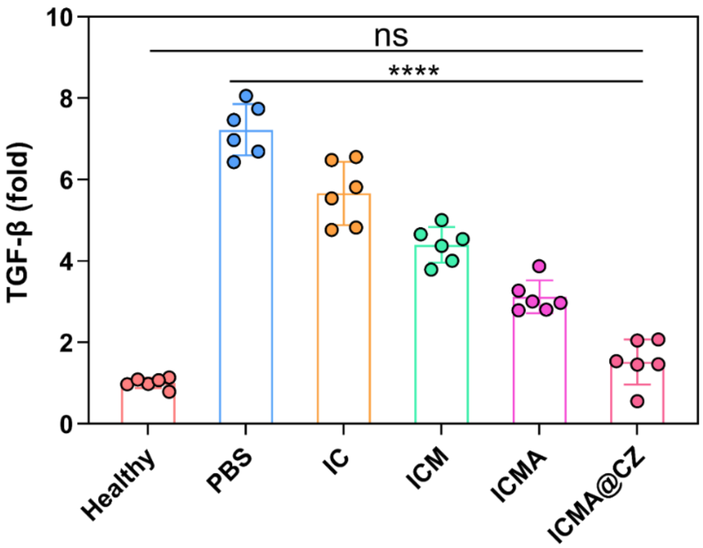

Fig. S18. Relative mRNA expression of TGF-β from the PBS, IC, ICM, ICMA or ICMA@CZ treatment groups ($n = 6$). Data were analyzed using Student's t-test (****$p < 0.0001$ *versus* PBS group).

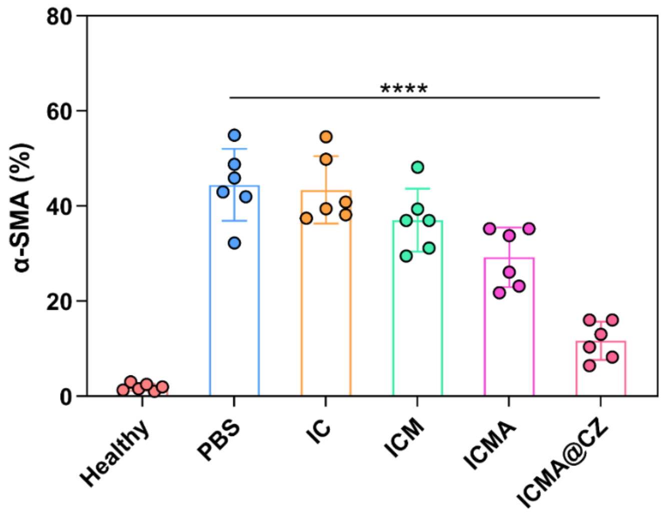

Fig. S19. Quantification analysis of α-SMA in CCl_4-induced fibrotic livers from the PBS, IC, ICM, ICMA or ICMA@CZ treatment groups was performed using Image J software (n = 6). Data were analyzed using Student's t-test (****$p < 0.0001$ *versus* PBS group).

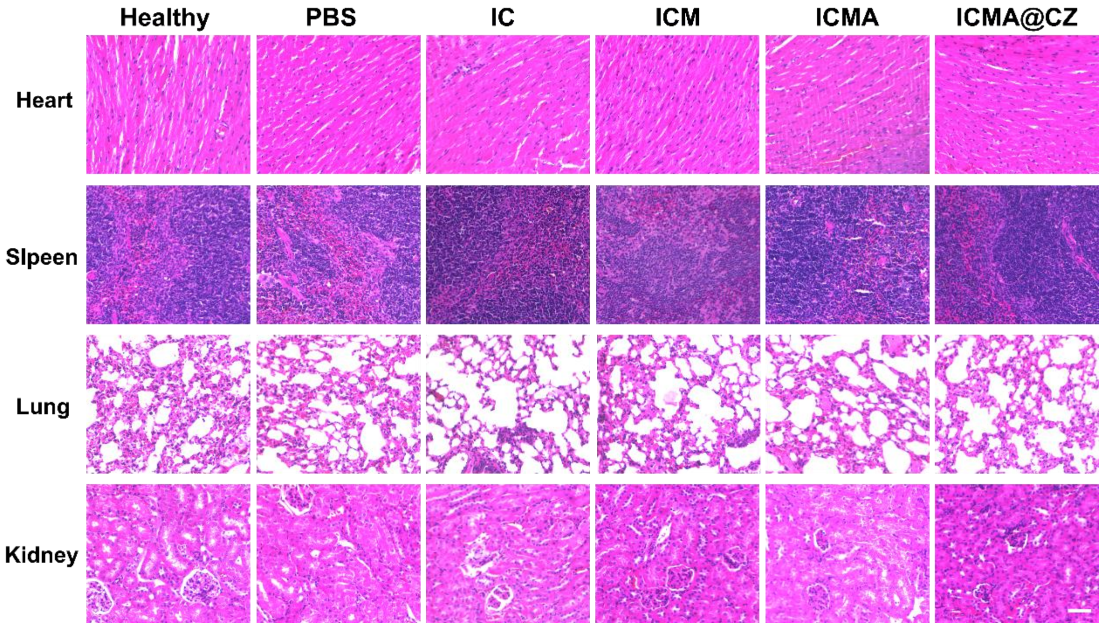

Fig. S20. H&E staining images of heart, spleen, lung and kidney from the mice treated with PBS, IC, ICM, ICMA or ICMA@CZ and sacrificed at day 36. Scale: 20 μm.

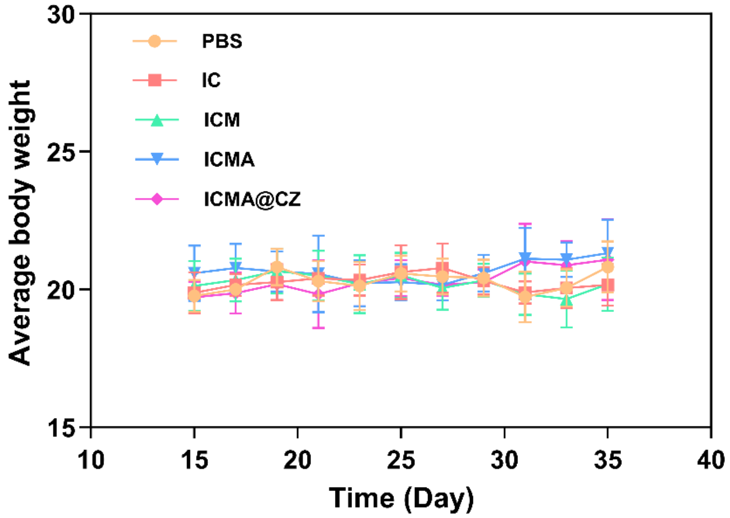

Fig. S21. The average body weight of the mice treated with PBS, IC, ICM, ICMA or ICMA@CZ at day 15, 17, 19, 21, 23, 25, 27, 29, 31, 33, 35.

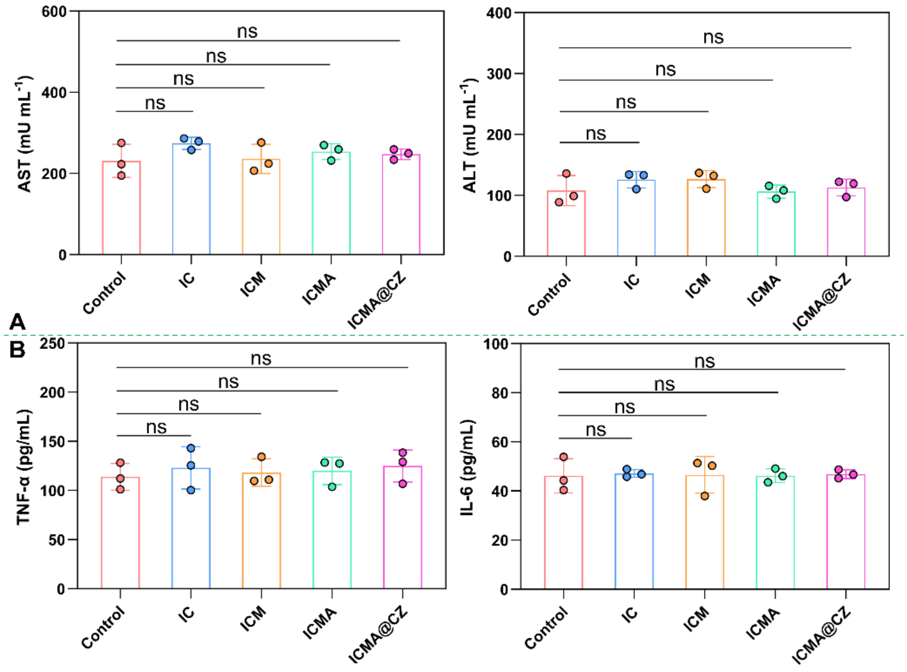

Fig. S22. ***In vivo* toxicity of nanosystems.** (**A**) Liver injury characterization by serum AST and ALT in healthy mice from the PBS, IC, ICM, ICMA or ICMA@CZ nanosystem treatment groups (n = 3); (**B**) Production of TNF-α and IL-6 (detected by ELISA) from the PBS, IC, ICM, ICMA or ICMA@CZ treatment groups (n = 3). Data were analyzed using Student's t-test.

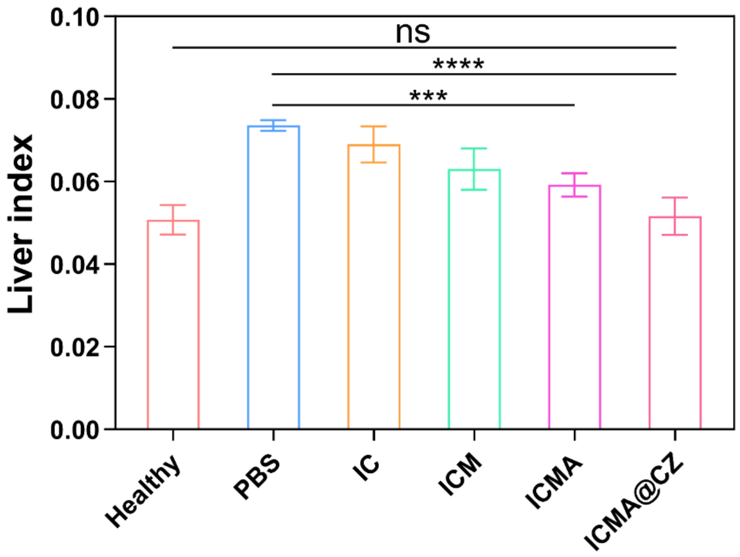

Fig. S23. Liver index of the mice treated with PBS, IC, ICM, ICMA or ICMA@CZ. Data were analyzed using Student's t-test (****$p < 0.0001$ *versus* PBS group, ***$p < 0.001$ *versus* PBS group).

Table S1. Primers used in this study.

**Primer**	**Sequence**
Mouse TNF-α Forward	CTA TGT CTC AGC CTC TTC TC
Mouse TNF-α Reverse	CAT TTG GGA ACT TCT CAT CC
Mouse IFN-β Forward	GCC TTT GCC ATC CAA GAG ATG C
Mouse IFN-β Reverse	ACA CTG TCT GCT GGT GGA GTT C
Mouse IL-6 Forward	GGC GGA TCG GAT GTT GTG AT
Mouse IL-6 Reverse	GGA CCC CAG ACA ATC GGT TG
Mouse Colla Forward	CGT ATC ACC AAA CTC AGA AG
Mouse Colla Reverse	GAA GCA AAG TTT CCT CCA AG
Mouse TGF-β Forward	GGA TAC CAA CTA TTG CTT CAC
Mouse TGF-β Reverse	TGT CCA GGC TCC AAA TAT AG
Mouse GAPDH Forward	CAC CAC CCT GTT GCT GTA GCC
Mouse GAPDH Reverse	ACC ACA GTC CAT GCC ATC AC
